# Bioactive Angucyclines/Angucyclinones Discovered from 1965 to 2023

**DOI:** 10.3390/md23010025

**Published:** 2025-01-05

**Authors:** Hai-Shan Liu, Hui-Ru Chen, Shan-Shan Huang, Zi-Hao Li, Chun-Ying Wang, Hua Zhang

**Affiliations:** School of Biological Science and Technology, University of Jinan, 336 West Road of Nan Xinzhuang, Jinan 250022, China; bio_liuhs@ujn.edu.cn (H.-S.L.); 202121201321@stu.ujn.edu.cn (H.-R.C.); huangshanshanyq123@163.com (S.-S.H.); 17863923880@163.com (Z.-H.L.); w2518885679@163.com (C.-Y.W.)

**Keywords:** angucyclines, angucyclinones, bioactivity, cytotoxicity, antimicrobial activity

## Abstract

Angucyclines/angucyclinones, a class of polyketides with diverse chemical structures, display various bioactivities including antibacterial or antifungal, anticancer, anti-neuroinflammatory, and anti-α-glucosidase activities. Marine and terrestrial microorganisms have made significant contributions to the discovery of bioactive angucyclines/angucyclinones. This review covers 283 bioactive angucyclines/angucyclinones discovered from 1965 to 2023, and the emphasis is on the biological origins, chemical structures, and biological activities of these interesting natural products.

## 1. Introduction

Nature has always been a significant source in the history of drug discovery, and the ocean has long been recognized as a reservoir of numerous lead compounds owing to its unique environment [1]. Over the past two decades, a substantial number of new marine natural products (MNPs) have been discovered [2]. Among them, over 45% of bioactive molecules sourced from microorganisms are produced by actinomycetes [3]. As the most well-known genus of actinomycetes, *Streptomyces* continues to yield a great diversity of novel bioactive compounds with varied chemical structures. Increasing evidence suggests that *Streptomyces* spp. are prolific producers of secondary metabolites with antibacterial/antifungal [4], anticancer [5], anti-neuroinflammatory [6], and anti-α-glucosidase [7] activities.

Angucyclines/angucyclinones are a class of polyaromatic polyketides that exhibit a huge diversity in chemical structures [8]. The first report of angucyclines/angucyclinones from *Streptomyces* species could date back to 1965 [9]. Angucyclines/angucyclinones could be isolated from both marine and terrestrial actinomycetes, especially *Streptomyces*. The decanone derived from acetyl-CoA is cyclized by polyketide cyclases to form the tetraene core of angucyclines/angucyclinones [10,11] with a characteristic angular benz[*α*]anthraquinone framework (the classical type) [12]. In certain instances, the typical angular tetracyclic angucyclines/angucyclinones undergo rearrangement into linear tetracyclic or tricyclic systems through enzymatic or non-enzymatic modifications, resulting in oxidized or rearranged benz[*α*]anthraquinone frameworks (the non-classical type) [13]. The oxidation state of the framework and the positions of substituents, combined with the presence of different types and numbers of sugars in *O*- and *C*-glycosides, contribute to the structural diversity of the angucyclines/angucyclinones [12]. Previous reviews published in 1992, 2012, and 2020 provided detailed and comprehensive summaries of their structures [10,12,14].

The structural diversity of angucyclines/angucyclinones confers upon them a variety of biological and pharmacological activities. The diverse chemical structures and biological activities of the compounds make them a focal point in drug discovery. Herein, the bioactive angucyclines/angucyclinones are categorized into compounds displaying both cytotoxic and antimicrobial activities, cytotoxic or antimicrobial activities only, and other activities, encompassing 283 angucyclines/angucyclinones discovered from 1965 to 2023. In this paper, the biological activities, chemical structures, and biological sources of these fascinating molecules are introduced.

## 2. Bioactive Angucyclines/Angucyclinones

**Figure 1:**
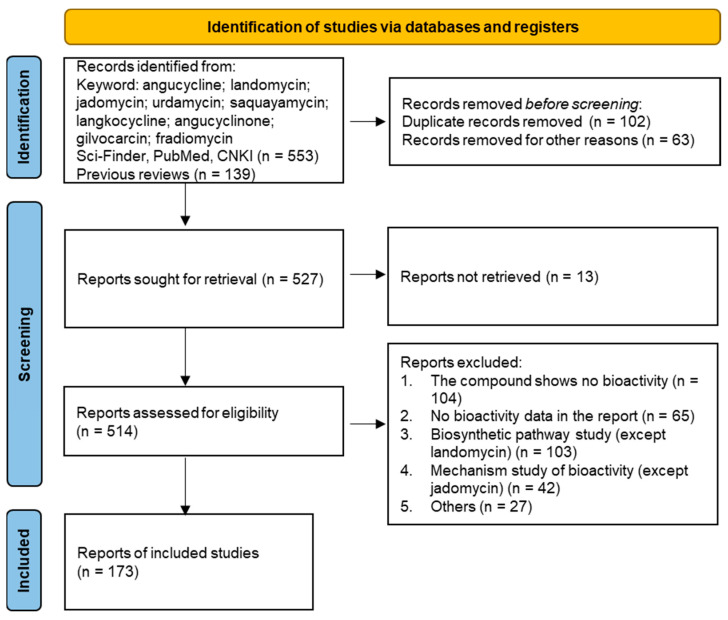
PRISMA 2020 flow diagram for systematic reviews.

The search for and screening of angucyclines/angucyclinones with bioactivity were conducted under the guidance of Preferred Reporting Items for Systematic reviews and Meta-Analyses (PRISMA) statement [15] (Figure 1). Cytotoxic activity with an IC_50_ value less than 10 μM, antibacterial or antifungal activity with an MIC value less than 128 μg/mL, and other activity that was comparable to or stronger than the positive control are considered as a bioactive compound.

### 2.1. Cytotoxic and Antibacterial or Antifungal Activities

#### 2.1.1. Marine-Derived Angucyclines/Angucyclinones

**Figure 2:**
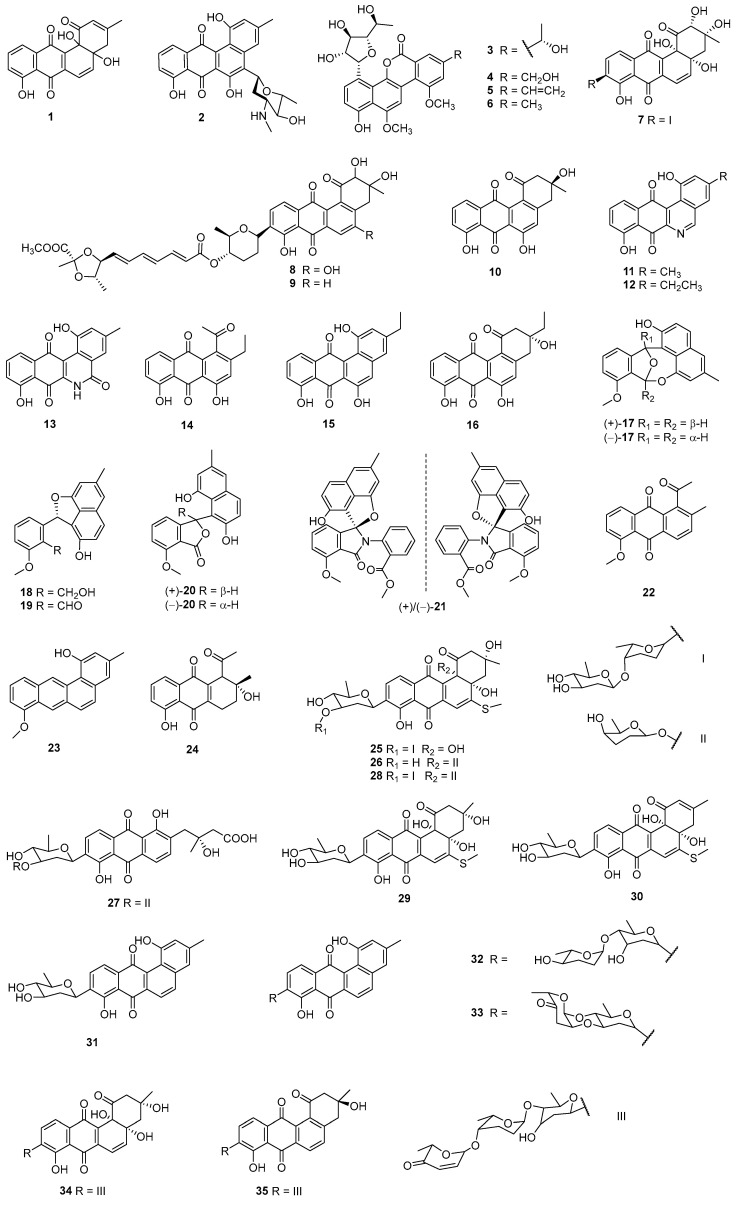
Structures of compounds **1**–**35.**

SS-228 Y (**1**) was obtained from sediment-derived *Chainia purpurogena* SS-228 (Sagami Bay, Japan) and exhibited various bioactivities. Compound **1** could prolong the survival period of mice inoculated with Ehrlich ascites tumor when the dosage was above 1.56 μg/piece/day in 10 days. Meanwhile, it showed broad-spectrum inhibition against Gram-positive bacteria with the minimum inhibitory concentrations (MICs) falling in the range of 0.78–12.5 μg/mL, except *Mycobacterium tuberculosis* [16] (Figure 2, Table 1). *Streptomyces* sp. HB202, which was isolated from the marine sponge *Halichondria panicea,* could generate a benz[a]anthracene mayamycin (**2**). Compound **2** exerted significant cytotoxic activities against HepG2 (hepatocellular carcinoma cells), HT-29 (colon cancer cells), GXF251L (gastric cancer cells), LXF529L (non-small-cell lung cancer cells), MAXF401NL (mammary cells), MEXF462NL (melanoma cells), PAXF1657L (pancreatic cancer cells), and RXF486L (renal carcinoma cells) with semi-inhibitory concentration (IC_50_) values in the range of 0.13–0.3 μM. Meanwhile, it could also inhibit *Bacillus subtilis* DSM 347, *Brevibacterium epidermidis* DSM 20660, *Dermabacter hominis* DSM 7083, *Klebsiella pneumoniae*, *Pseudomonas aeruginosa* DSM 50071, *Staphylococcus aureus* ATCC 12600, *S. epidermidis* DSM 20044, and *S. lentus* DSM 6672, with the IC_50_ values within 0.31–8.4 μM, comparable to the positive control chloramphenicol [17]. A 3 m deep-soil-derived *Streptomyces* sp. QD01-2 collected in Qingdao, China, produced gilvocarcin HE (**3**) together with gilvocarcins H (**4**), V (**5**), and M (**6**), and all of them showed antibacterial activities against *S. aureus*, *B. subtilis*, *Escherichia coli,* and *Candida albicans* with MIC values of 0.1–25 μM. Gilvocarcin V (**5**) was cytotoxic to MCF-7 (breast cancer cells), K562 (leukemic cells), and P388 (mouse leukemia cells) with IC_50_ values ranging within 0.8–1.8 μM [18]. The marine *S. fradiae* PTZ0025 was the producer of the fradimycins A (**7**), B (**8**), and MK844-mF10 (**9**), which exhibited inhibition of human colon cancer HCT-15 (colon cancer cells), SW620 (colon cancer cells), and rat glioma C6 cells (IC_50_ values = 0.13–6.46 μM) as well as *S. aureus* (MICs = 6.0, 2.0, and 4.0 μg/mL, respectively) [19].

Rabelomycin (**10**) and phenanthroviridone (**11**) were isolated from the culture of *Micromonospora rosaria* SCSIO N160, which was obtained from a sediment sample from the South China Sea. Both of them could inhibit SF-268 (neurocarcinoma cells), MCF-7, and NCI-H460 (large-cell lung cancer cells) with IC_50_ values of 0.09–9.91 μM, and they also displayed antibacterial activities against *E. coli* ATCC 25922, *S. aureus* ATCC 29213, *B. thuringiensis* SCSIO BT01, and *B. subtilis* SCSIO BS01 with MICs of 0.25–60 μg/mL [20]. The marine-derived *Micromonospora echinospora* SCSIO 04089 generated homophenanthroviridone (**12**), homophenanthridonamide (**13**), nenesophanol (**14**), rabelomycin E (**15**), and homorabelomycin (**16**), which exhibited activities against cancer cells and pathogenic bacteria or fungi. Compound **12** could inhibit SF-268, MCF-7, and HepG2 cells with IC_50_ values ranging from 1.4 to 5.4 µM, and the IC_50_ values of **14** and **16** ranged from 7.6 to 12.5 µM, while **13** could inhibit only HepG2 cells (IC_50_ = 4.0 µM). Compound **12** also displayed inhibition to *S. aureus* ATCC 29213, *B. thuringensis* SCSIO BT01, *B. subtilis* 1064, *M. luteus* SCSIO ML01, and methicillin-resistant *S. aureus* (MRSA) shhs-A1, and their MICs ranged from 2 to 4 μg/mL, while **15** could inhibit *S. aureus* ATCC 29213 and *M. luteus* SCSIO ML01 at the concentrations of 4 and 8 μg/mL, respectively [21].

(±)-Actinoxocine (**17**), actinaphthorans A (**18**) and B (**19**) [22], as well as (±)-pratenone A (**20**) [23], were isolated from *S. pratensis* KCB-132, which was associated with sediment collected in Jiaozhou Bay, China. Compound **17** is characterized by a unique epoxybenzo[*f*]naphtho[1,8-bc]oxocine carbon skeleton. Compounds **18** and **19** were two unusual C-ring cleavage analogues with cytotoxicities and antibacterial activities against human colon cancer cells LS180 (IC_50_ values = 1.9 μM), *B. cereus* (MIC = 2 μg/mL), and *Colletotrichum lagenarium* (MIC = 2 μg/mL). Enantiomers of **17** could inhibit various bacteria and fungi with MICs of 8–32 μg/mL [22], while (±)-pratenone A (**20**) revealed antibacterial activity against *S. aureus* CMCC 26003 with an MIC of 8 μg/mL [23]. Further study of *Streptomyces* sp. KCB-132 led to the isolation of the nitrogen-containing enantiomers (±)-pratensilin D (**21**) and compound **22**, featuring an A-ring cleavage structural property. Compound (–)-**21** exhibited moderate cytotoxicity to the NCI-H460 and HepG2 cell lines, with respective IC_50_ values of 4.6 and 9.3 µg/mL, while (+)-**21** was active only against NCI-H460 cells (IC_50_ = 9.2 µg/mL). Compound **22** displayed cytotoxicities to colon 38 (colon cancer) and HeLa (cervical cancer) cells, with IC_50_ values of 7.3 and 10.3 µg/mL, respectively. Compound (–)-**21** also exhibited selective inhibitory activity against *B. cereus* CMCC 32210 with an MIC value of 4 µg/mL, while (+)-**21** showed no efficacy against all tested microbial strains, up to 64 µg/mL [24]. The culture extract of *Streptomyces* sp. KCB-132 also contained an antibiotic compound actetrophenol A (**23**), which displayed moderate activities against Gram-positive strains with MICs ranging within 1–16 μg/mL. Meanwhile, **23** also showed inhibition toward multiple resistant strains, especially *S. aureus* and *Enterococcus faecium*, with an MIC of 4 μg/mL, better than the positive control, penicillin (MIC > 32 μg/mL) [25].

The research group of **23** also discovered **24,** from *S. pratensis* KCB-132, with moderate activities against multiple resistant “ESKAPE” pathogens (*E. faecium*, *S. aureus*, *K. pneumoniae*, *Acinetobacter baumannii*, *P. aeruginosa*, and *Enterobacter* species); the MICs of **24** ranged within 3.1–21.4 μg/mL, comparable to the positive controls ampicillin, amikacin, and ciprofloxacin [26]. *S. ardesiacus* 156VN-095 was isolated from a sample collected near Nha Trang Bay, Vietnam, and the fermentation extract contained urdamycins W (**25**) and X (**26**), grincamycin U (**27**), as well as an analogue, urdamycin E (**28**). Compounds **25**, **26**, and **28** were cytotoxic to ACHN (renal adenocarcinoma cells), HCT-15, MDA-MB-231 (breast cancer cells), NCI-H23 (non-small-cell lung cancer cells), NUGC-3 (gastric cancer cells), and PC-3 (prostate cancer cells) with GI_50_ (50% growth inhibition concentration) values of 0.019–0.150 μM, comparable to those (0.140–0.162 μM) of Adriamycin. In addition, **25**–**27** displayed antibacterial activities against *B. subtilis* KCTC 1021, *Micrococcus luteus* KCTC 1915, and *S. aureus* KCTC 1927, and the MICs ranged from 8 to 64 µg/mL [27]. Marine-derived *Streptomyces* sp. BCC45596 collected from Sichang Island (5 m deep, Chonburi province, Thailand) generated C-glycosylated benz[*α*]anthraquinone urdamycinone E (**29**), urdamycinone G (**30**), dehydroxyaquayamycin (**31**), and urdamycin E (**28**). Compounds **29**–**31** displayed inhibition against KB (oral epidermoid cancer cells), MCF-7, NCI-H187 (retinoblastoma cells) and Vero (African green monkey kidney cells) (IC_50_ values = 0.092–15.46 μg/mL); *M. tuberculosis* (IC_50_ values = 3.13–12.50 μg/mL) as well as *Plasmodium falciparum* (IC_50_ values = 0.0534–22.93 μg/mL) [28]. *Streptomyces* sp. SCSIO 11594 was isolated from a 2403 m deep sediment sample collected from the South China Sea, and the fermentation broth contained marangucyclines A (**32**) and B (**33**). The ketose-containing compound **33** exhibited inhibition against A594 (non-small-cell lung cancer cells), CNE2 (parotid cyst cancer cells), HepG2, and MCF-7, and the IC_50_ values ranged from 0.24 to 0.56 μM; it also displayed selectivity between cancer cells and normal cells. Marangucyclines A (**32**) and B (**33**) also showed weak antibacterial activities against *E. faecalis* ATCC29212 (both MICs at 64 μg/mL) [29]. *S. lusitanus* SCSIO LR32 was isolated from a deep-sea sediment sample collected in the South China Sea, China. A-7884 (**34**) and grincamycin J (**35**), produced by SCSIO LR3, exhibited cytotoxic activities against human cancer cells MDA-MB-435 (melanoma), MDA-MB-231, NCI-H460, HCT-116 (colon cancer), and HepG2 as well as MCF-10A (normal breast epithelial cells) with IC_50_ values ranging from 0.4 to 6.9 µM. In addition, A-7884 (**34**) demonstrated antimicrobial activity against *M. luteus*, with an MIC value of 1.95 µg/mL [30].

#### 2.1.2. Terrestrial-Derived Angucyclines/Angucyclinones

**Figure 3:**
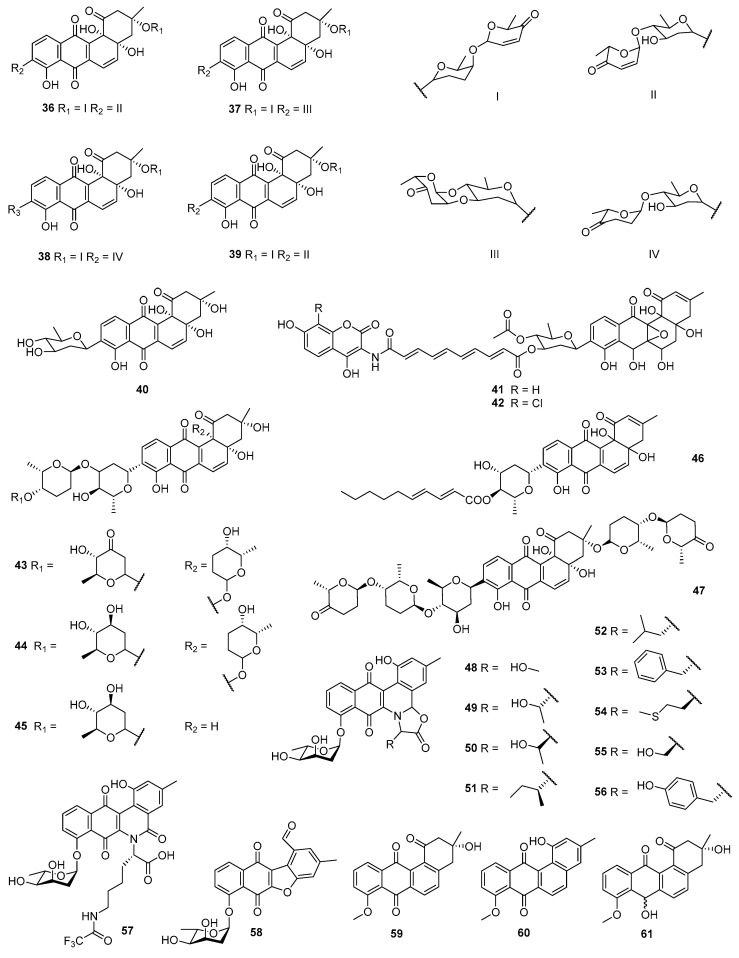
Structures of compounds **36**–**61.**

The fermentation of *S. gilvotanarens* NRRL 11382, a new species isolated from a soil sample collected in Kochi, Japan, led to the discovery of gilvocarcins V (**5**) and M (**6**). Both were bioactivated toward tumor cells such as sarcoma 180 (mouse malignant sarcoma cells) and P388. Mice treated with compound **5** lived significantly longer than control animals. Compounds **5** and **6** also showed inhibition against *S. aureus* ATCC 6538P and *B. subtilis* No. 10707, with MICs of 0.05/1.6 and 0.78/25 μg/mL, respectively [31]. Saquayamycins A–D (**36**–**39**) were produced by *S. nodosus* MH190-16F3, associated with a soil sample taken from tobacco growth areas (Kitakyushu, Japan), and could inhibit Adriamycin-sensitive (P388/S) and Adriamycin-resistant (P388/ADR) sublines of P388 in vitro, with IC_50_ values of 0.06–0.15 μg/mL. The LD_50_ (median lethal dose) by intraperitoneal injection of saquayamycins A (**36**) and B (**37**) in mice were 6.25–12.5 mg/kg. In addition, **36**–**39** exhibited antibacterial activities against *S. aureus* FDA209P, *M. lysodeikticus* IFO 3333, *M. luteus* PCI1001, and *B. subtilis* PCI 219 with MICs of 1.56–6.25 μg/mL [32] (Figure 3). Further research revealed that saquayamycins A (**36**) and B (**37**) exhibited remarkable activities against L-1210 (mouse leukemia cells), A549, and HT-29 (IC_50_ values = 0.004, 0.2, and 0.06 μg/mL, respectively), and demonstrated distinct toxicity in vivo [33]. Aquayamycin (**40**) and Adriamycin (positive control) could also inhibit the aforementioned two cells with IC_50_ values at 2.0/2.2 and 0.01/0.55 μg/mL, respectively [32]. *S. antibioticus* Tü 6040 was isolated from a soil sample from Iguaguu, Argentina, and the mycelium extract contained the simocyclinones D4 (**41**) and D8 (**42**), both of which showed cytotoxicities and antibacterial activities, while the GI_50_s against HMO2 (human milk oligosaccharides) and MCF-7 cell lines ranged from 0.3 to 5.6 μM, better than 5-fluorouracil (positive control, GI_50_ = 1.2 and 50 μM). The MICs against *B. brevis* DSM30 were 30 and 10 μg/mL, respectively [34,35].

Kerriamycins A–C (**43**–**45**) (produced by *S. violaceolatus*) [36] and capoamycin (**46**) (produced by soil-derived *S. capoamus* collected in Fujioka, Japan) [37], which were discovered by the same research group, could prolong the survival periods of mice bearing Ehrlich ascites carcinoma when they were subjected to intraperitoneal injections on days 1 and 5. The LD_50_ of **46** was 15 mg/kg (ip), and the antitumor activity was based on an induction effect on the differentiation process of mouse myeloid leukemia cells (M1). Meanwhile, **43**–**46** displayed inhibitory activities against *S. aureus* FDA 209P, *B. subtilis* ATCC 6633, *B. cereus* IAM 1729, and *M. luteus* ATCC 9341, with the MICs at 1.65–25 μg/mL. In addition, **46** showed activity against *Penicillium chrysogentrrn* ATCC 10002 and *Trichophyton mentagrophytes*, and the MICs were 1.56 and 12.5 μg/mL, respectively. Grincamycin (**47**), produced by *S. griseoincarnatus*, was also isolated by the same group above and was revealed to exert significant cytotoxicity toward the P388 cell line (IC_50_ = 13 ng/mL) and moderate antibacterial activities against *S. aureus* FDA 209P (MIC = 50 μg/mL), *M. luteus* ATCC 9341 (MIC = 25 μg/mL), and *B. cereus* IAM 1729 (MIC = 50 μg/mL) [38].

The fermentation of *S. venezuelae* ISP5230 led to the discovery of jadomycins S (**48**), T (**49**), DT (**50**), B (**51**), L (**52**), F (**53**), DM (**54**), DS (**55**), and Y (**56**), with cytotoxicities and antibacterial activities. Compounds containing L-serine and D/L-threonine displayed higher cytotoxicities against MDA-MB-435; the IC_50_ values of **48**–**50** ranged from 1.06 to 2.82 μM. Jadomycins containing aromatic side chains showed the lowest activities against T-47D (breast cancer cells), indicating that the activities of **48**–**50** are related to the hydrogen donor of the hydroxyl side chain. All these jadomycins (**48**–**56**) displayed inhibitory activities against *S. aureus* C622 (ATCC 25923), *S. aureus* 305, *S. aureus* BeckerCP8 (ATCC 49525), *S. aureus* BeckerLyc12CP336 (ATCC 55804), *S. epidermidis* C960 (ATCC 14990), *S. epidermidis* C621 (clinical isolate), and *B. subtilis* C971 (ATCC 6633), with MICs of <1–64 μg/mL. Especially, the MICs against *S. aureus* C623 (MRSA) were all less than 1–8 μg/mL, better than that of the positive control erythromycin (MIC > 128 μg/mL) [39]. Jadomycins retain their cytotoxic properties toward multi-drug-resistant (MDR) breast cancer cells because they cannot be expelled by ATP-binding cassette (ABC) transporters, which is an important reason for tumor resistance to doxorubicin [40]. Further study confirmed that the cytotoxicities of jadomycins **48** and **51**–**53** were minimally affected by the efflux transporter functions of ABCB1, ABCC1, and ABCG2 [41]. Jadomycin DS (**55**) could bind to a variety of proteins, likely in a non-specific manner. The quality and quantity of direct binding between topoisomerase IIβ and jadomycin DS (**55**) were demonstrated by WaterLOGSY NMR spectroscopy [42]. The addition of *N*_ε_-trifluoroacetyl-_L_-lysine in the fermentation of *S. venezuelae* ISP5230 led to the isolation of **57** and **58,** containing amide and furan rings. The oxazolone-ring-containing **57** was active against MRSA (MIC = 3 μg/mL), *S. warneri* (3 μg/mL), and vancomycin-resistant *Enterococcus faecium* (VRE, 13 μg/mL). In contrast, **58** was much less active (MIC ≥ 100 μg/mL) against the three Gram-positive strains. The enhanced antibiotic activity of **57** in comparison with **58** implies that the hemiaminal ether functionality plays an important role in the antimicrobial properties of the jadomycins [43]. *Streptomyces* sp. AC113 was isolated from the root of *Taxus chinensis* (Bakata) and could produce (–)-8-*O*-methyltetrangomycin (**59**), 8-*O*-methyltetrangulol (**60**), and 8-*O*-methyl-7-deoxo-7-hydroxytetrangomycin (**61**). These three compounds were cytotoxic to mouse melanoma B16 (IC_50_ = 0.054–7.13 μg/mL) and HT-29 (IC_50_ = 8.59–66.9 μg/mL) cells, and they showed antibacterial activities against *P. aeruginosa* CCM 3955, *S. aureus* CCM 3953, *E. coli* CCM 3988, *L. monocytogenes* NCTC 4886, *B. subtilis* CCM 2216, and *B. cereus*, with MIC values ranging from 0.6 to 78.6 μg/mL [44].

In addition to fermenting the producer of jadomycins in the presence of amino acid analogues, semi-synthesis, structural gene deletion, and deletion or heterologous expression of sugar biosynthetic genes led to the discovery and isolation of more than 70 jadomycins [45]. This enabled a comprehensive evaluation of the cytotoxic and antibacterial activity of jadomycins and facilitated the study of the mechanism of bioactivity [46]. The cytotoxicity of jadomycins involves the generation of cytosolic superoxide via a Cu(II)–jadomycin reaction, a mechanism common to all the jadomycins tested and observed in MCF7-CON and drug-resistant MCF7-TXL cells. The generation of intracellular ROS in the superoxide dismutase 1, glutathione, and peroxiredoxin/thioredoxin cellular antioxidant enzyme pathways was scavenged by jadomycin treatment. The blocking of these antioxidant pathways may enhance the cytotoxic potency of jadomycin in both drug-sensitive and drug-resistant breast cancers [47]. The breast cancer cell death induced by jadomycins is independent of ROS activity through the inhibition or poisoning of type II topoisomerases and the induction of DNA damage and apoptosis, and jadomycins B (**51**) and F (**53**) selectively poison topoisomerase IIb to induce DNA damage and apoptosis. [48]. The pharmacokinetics, toxicities, and antitumoral effects in zebrafish larvae and mice showed that jadomycin B (**51**) had a good safety profile and provided partial antitumoral effects [49] together with the generation of reactive oxygen species (ROS) induced by copper [47], the inhibition of topoisomerase IIα and IIβ [48], and the avoidance of ABC transporters [40]. All these observations suggest that jadomycins may be used as a breast cancer chemotherapy in clinical practice, while further studies on their ability to penetrate the blood–brain barrier are required [50].

**Figure 4:**
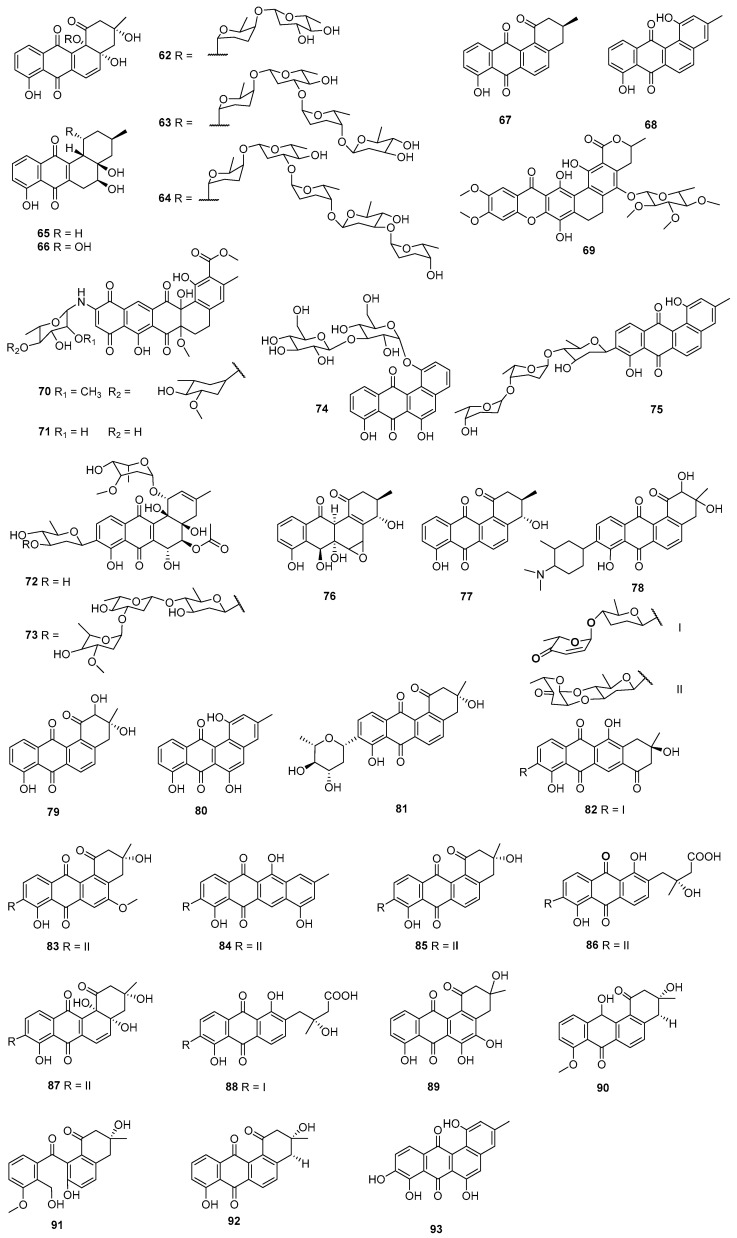
Structures of compounds **62**–**93.**

Langkocyclines A1–A3 (**62**–**64**) were obtained from the extract of *Streptomyces* sp. Acta 3034, which was associated with the rhizospheric soil of *Clitorea* sp. Compound **64** displayed inhibitory activities against HepG2 and NIH 3T3 (mouse embryonic fibroblast) cells, with IC_50_ values of 2.5–5.0 μM, while **62**–**64** showed inhibition toward *B. subtilis*, with IC_50_ values at 40.7, 4.07, and 2.17 μM, respectively [51] (Figure 4). The fermentation of soil-derived *Saccharopolyspora* BCC 21906 (Chanthaburi, Thailand) led to the isolation of saccharosporones A (**65**) and B (**66**), as well as (+)-ochromycinone (**67**) and tetrangulol methyl ether (**68**). Compounds **65** and **66** exhibited cytotoxic activities against KB, MCF-7, and NCI-H187 cell lines with IC_50_ values ranging within 3.4–9.1µM. Compounds **65**–**68** showed growth inhibition against *M. tuberculosis*, with IC_50_ values of 76.2, 72.7, 40.8, and 19.7 µM, respectively [52]. Heterologous expression of two eDNA-derived KSβ sequences associated with the biosynthesis of (C24)-pradimicin and (C26)-xantholipin-type metabolites in *Streptomyces salbus* led to the isolation of calixanthomycin A (**69**) and the arenimycins C (**70**) and D (**71**), and all of them were cytotoxic toward HCT-116 cells, with respective IC_50_ values at 0.43 nM, 0.17 μM, and 2.8 μM. Meanwhile, **69**–**71** also could inhibit MRSA and *B. subtilis* RM125 with MICs of 0.0015–50 μg/mL [53]. The overexpression in *S. chattanoogensis* L10 (CGMCC 2644) of a pathway-specific activator gene under the constitutive *erm*E* promoter successfully triggered the expression of the angucycline biosynthetic genes and led to the discovery of chattamycins A (**72**) and B (**73**). Compound **72** was cytotoxic to MCF-7 (IC_50_ = 6.46 μM), while **73** showed inhibitory activities against MCF-7 (IC_50_ = 1.08 μM) and HepG2 (IC_50_ = 5.93 μM) cells. In addition, **73** exhibited activity against *B. subtilis* ATCC 67736 (IC_50_ = 102.59 μM) [54]. The method of site-directed mutagenesis led to the generation of ten mutants of *S. chattanoogensis* L10 (CGMCC 2644) with point mutations in the highly conserved region of rpsL (encoding the ribosomal protein S12) or rpoB (encoding the RNA polymerase β-subunit). L10/RpoB (H437Y) accumulated anthrachamycin (**74**), which was absent in the wild type. In the 2,2′-amino-di(2-ethyl-benzothiazoline sulfonic acid-6) ammonium salt (ABTS) free radical scavenging assay and the ferric ion reducing antioxidant power (FRAP) iron reduction assay, **74** showed antioxidant activity at 67.28 and 24.31 mg VCE/g LP, respectively [55].

C-glycosylated benz[*α*]anthraquinone, dehydroxyaquayamycin B (**75**) was isolated from the fermentation broth of *S. blastomycetica* F4-20 associated with the root of *Tripterygium wilfordii* Hook. f. Compound **75** showed cytotoxic activities against the BGC823 (gastric adenocarcinoma) and HeLa cell lines with IC_50_ values of 0.71 and 1.34 μg/mL, respectively, and it also displayed antifungal activities against *Valsa mali*, *C. orbiculare*, and *Fusarium graminearum* at 50 μg/mL with inhibition rates of 41.5%, 58.3%, and 51.0%, respectively [56]. *S. bulli* GJA1, associated with *Gardenia jasminoides*, was the producer of **76** and **77**, both of which were cytotoxic toward OV90 and ES2 ovarian cancer cells with MICs of 0.36/0.55 µM and 2.42/1.69µM, respectively, better than paclitaxel and cisplatin. Compound **76** also showed antivirulence activity by inhibiting the phenol-soluble modulin (PSM) production and the biofilm formation of MRSA [57]. Strain NJES-13T, a newly established actinobacteria genera *Aptenodytes* in the family *Dermatophilaceae*, was isolated from the gut microbiota of the Antarctic emperor penguin. The fermentation broth of NJES-13T contained 2-hydroxy-frigocyclinone (**78**) and 2-hydroxy-tetrangomycin (**79**). Compound **78** showed inhibitory activities against HL-60 (leukemia), Bel-7402 (hepatocellular carcinoma), and A549 cells, with IC_50_ values ranging from 4.2 to 8.5 µM, while **78** and **79** could inhibit *S. aureus*, *B. subtilis*, and *C. albicans* with MICs of 5.7–27.2 µg/mL [58]. A soil-derived *S. cellulosae* YIM PH20352 (Yunnan province, China) produced rabelomycin (**10**), dehydrorabelomycin (**80**) [59], urdamycinone B (**81**) and dehydroxyaquayamycin (**31**) [60]. Compounds **10** and **80** could inhibit the root rot pathogens of *Panax notoginseng,* including *Plectosphaerella cucumerina*, *Alternaria panax*, *F. oxysporum*, and *F. solani*, with MICs of 32–128 μg/mL. Compound **81** exhibited antifungal activities against *A. panax* and *P. cucumerina* with MICs at 16 and 64 μg/mL, respectively, and **31** showed inhibitory activity toward *A. panax* with the MIC at 64 μg/mL. *Streptomyces* sp. XZHG99T was isolated from a soil sample collected from the Color desert (Tibet Autonomous Region, China), and produced grincamycins L–N (**82**–**84**) as well as the known compounds rabelomycin (**10**), moromycin B (**85**), fridamycin D (**86**), and saquayamycin B1 (**87**), all of which showed inhibitions against A549, H157 (non-small-cell lung cancer), MCF-7, MDA-MB-231, and HepG2 cells with the IC_50_ values ranging from 1.52 to 17.3μM. Compound **10** also exhibited antibacterial activities toward *Mycolicibacterium smegmatis* and *S. aureus*, with IC_50_ values from 0.12 to 23.1 μM [61].

*Streptomyces* sp. IB201691-2A, which was obtained from the endemic mollusk *Benedictia baicalensis* of Lake Baikal, was the producer of baikalomycin C (**88**), rabelomycin (**10**), and 5-hydroxy-rabelomycin (**89**). Baikalomycin C (**88**) displayed inhibition of Huh7.5 (hepatocellular carcinoma) and SW620 cells (IC_50_ values = 7.62 and 3.87 µM, respectively) as well as *S. carnosus* DSMZ 20501 (MIC = 62 µM), while **10** and **89** showed inhibitory activities against A549, Huh7.5, and SW620 cells (IC_50_ values = 7.21–13.43 µM) as well as *Erwinia persicina* DSMZ 19328, *S. carnosus* DSMZ 20501, and *M. smegmatis* DSMZ 43286 (MICs = 31–125 µM) [62]. 12-Deoxo-12-hydroxy-8-*O*-methyltetrangomycin (**90**), the C-ring cleavage product of angucyclinone C (**91**), tetrangomycin (**92**), and 8-*O*-methyltetrangomycin (**59**) were isolated from the secondary metabolites of *Streptomyces* sp. CB01913 (soil sample of Weishan County, Yunnan Province, China). Compounds **90**, **92**, and **59** exhibited inhibitory activities against SF295 (malignant glioma cells) and H226 (lung squamous cells) with the IC_50_ values ranging from 3.1 to 10 μM. Compounds **92** and **59** also inhibited M14 (melanoma cells) with IC_50_ values of 2.4 and 9.7 μM, respectively. Meanwhile, **91**, **92**, and **59** displayed antibacterial activities toward *S. aureus* ATCC 25923, *B. subtilis* ATCC 23857, and *M. smegmatis* ATCC 607, with the MIC values ranging from 8.1 to 93 μg/mL [63]. 6,9-Dihydroxytetrangulol (**93**) was isolated from *S. lividans* TK23 transformed with a kinanthraquinone biosynthetic gene cluster in which the *kiqO* gene was disrupted. Compound **93** revealed both cytotoxicity and antibacterial activity; the IC_50_ toward HL-60 cells was 5.1 μM, and the IC_50_ values toward *S. aureus* and *C. albicans* were 1.9 and 1.1 μM, respectively, better than chloramphenicol [64].

#### 2.1.3. Angucyclines/Angucyclinones from Other Sources

**Figure 5:**
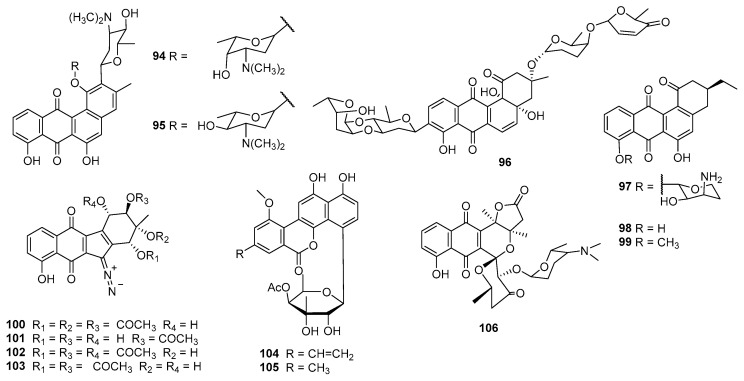
Structures of compounds **94**–**106.**

*Nocardia lurida* was the producer of benzanthrins A (**94**) and B (**95**), which exhibited antibacterial activities against various Gram-positive bacteria, with MIC values between 0.2 and 3.1 μg/mL [65], and cytotoxicities against 9KB (nasopharyngeal carcinoma cells) and 9PS (IC_50_ values = 0.3 and 0.01 μg/mL, respectively) (Figure 5). It was interesting that benzanthrin A (**94**) caused a reversal of adenosine cyclic 3′,5′-monophosphate-induced morphological changes in AC glioma tumor (9ASK) cells at 10 μg/mL, while no reversal was observed with benzanthrin B (**95**) [66]. The fermentation broth of *S. matensis* A-6621 contained PI-083 (**96**), which exhibited cytotoxicity against the KB cell line with the IC_50_ at 0.026 μM and inhibitory activities toward *S. aureus* 209P-JC, *Sepidermidis* IID 866, *E. faecium* ATCC 8043, *B. cereus* S 1101, and *B. subtilis* ATCC 6633 with the MICs at 0.39, 1.56, 3.13, 12.5, and 1.56 μg/mL, respectively [67]. Brasiliquinones A–C (**97**–**99**), which were isolated from the culture broth of the pathogenic *Nocardia* sp. IFM 0089, displayed inhibitory activities toward the L-1210 and P388 cell lines (IC_50_ values = 2.9–7.0 μg/mL) and were also active against P388/ADR cells, with IC_50_ values ranging from 3.0 to 3.8 μg/mL. Compounds **97**–**99** also showed antibacterial activities against *S. aureus* 209P, *S. aureus* MRSAIFM 62971, *M. smegmatis* ATCC 607, and *M. luteus* IFM 2066, with the MICs ranging from 0.39 to 50 μg/mL [68].

Kinamycins A–D (**100**–**103**), isolated from *S. murayamaensis,* have a highly unusual and potentially reactive diazo group. Kinamycins A (**100**) and C (**102**) showed IC_50_ values of 10 μM and 0.3 μM, respectively, against Chinese hamster ovary (CHO) cancer cells. Kinamycins A (**100**) and C (**102**) also could inhibit the catalytic decatenation activity of DNA topoisomerase IIα, but showed no activity as a topoisomerase II poison. Meanwhile, their inhibition of catalytic activity was not correlated with a cell growth inhibitory effect [69]. Kinamycins A–D (**100**–**103**) also showed antibacterial activities against Gram-positive bacteria [70]. 4′-acetylated-chrysomycins A (**104**) and B (**105**) were discovered during the screening for antitumor agents from the metabolites of actinomycetes, and both compounds showed high cytotoxicities toward most of the tested cancer cells, with IC_50_ values less than 10 ng/mL. Compound **104** showed strong anti-Gram-positive-bacterial activities toward MRSA and VRE, with MIC values of 0.5–2 μg/mL [71]. *S. aureofaciens* CCM 3239, received from the Czech Collection of Microorganisms (CCM, Brno, Czech Republic), produced auricin (**106**) with cytotoxicities against the human ovarian carcinoma cell line A2788 (IC_50_ = 1.05 μM), cisplatin-resistant cells A2780/CP (IC_50_ = 0.7 μM), MDA-MB-231 (IC_50_ = 4.19 μM), and MCF-7 (IC_50_ = 2.8μM). Compound **106** was active against *B. subtilis* and *S. aureus* Newman, with MICs at 4.6 and 9.2 μM, respectively [72].

### 2.2. Cytotoxicities

#### 2.2.1. Marine-Derived Angucyclines/Angucyclinones

**Figure 6:**
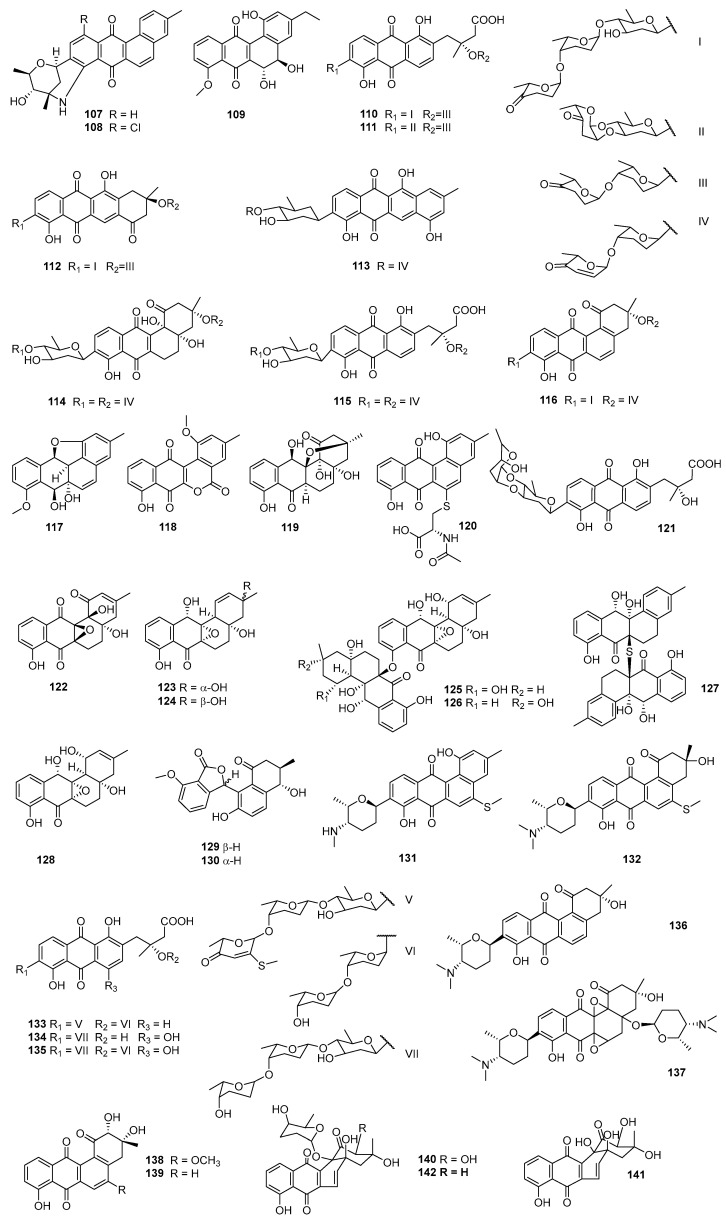
Structures of compounds **107**–**142.**

A sediment-derived actinomycete, *Streptomyces* CNH990, produced marmycins A (**107**) and B (**108**), which exhibited cytotoxicities against HCT-116 cells with IC_50_ values at 60.5 and 1.09 μM, respectively. For marmycin A (**107**), tumor cell cytotoxicity appeared to coincide with the induction of modest apoptosis and arrest in the G_1_ phase of the cell cycle [73] (Figure 6). A sponge-derived *Saccharopolyspora taberi* PEM-06-F23-019B (Tanzanian) produced PM070747 (**109**), which displayed cytotoxicities against MDA-MB-231, HT-29, and A549 cells with the IC_50_ values at 0.71, 1.42, and 3.28 μM, respectively [74]. The secondary metabolites of *S. lusitanus* SCSIO LR32 contained grincamycin (**47**); grincamycins B (**110**), C (**111**), and E (**112**) [75]; grincamycins H–J **(113**, **116,** and **35)**, congers P-1894B (vineomycin A1, **114**), saquayamycin B (**37**) [29], vineomycin B2 (**115**), and A-7884 (**34**) [30]. Grincamycins B (**110**), C (**111**), and E (**112**) and grincamycin (**47**) displayed cytotoxicities against the B16 and HepG2 cell lines with IC_50_ values of 1.1–11 μM. Compounds **110** and **47** could inhibit SW-1990 (pancreatic cancer) and HeLa cell lines with IC_50_ values of 5.4–11 μM [75], while **37** and **113**–**115** showed inhibitory activities against Jurkat T (acute T-cell leukemia cells) with IC_50_ values of 0.011–3.0 μM (positive control, doxorubicin, 0.034 μM) [13]. Grincamycins I (**116**), J (**35**), and A-7884 (**34**) were cytotoxic to tumor cells MDA-MB-435, MDA-MB-231, NCI-H460, HCT-116, and HepG2, with the IC_50_ values at 0.4–6.9 µM, and they also showed toxicity to the normal cells MCF-10A with IC_50_ values of 22.43–2.90 µM [30]. Meanwhile, saquayamycin B (**37**), which was isolated from an intertidal sediment-derived *Streptomyces* sp., displayed significant cytotoxicities against HepG2, SMMC-7721 (hepatocellular carcinoma cells), and PLC-PRF-5 (hepatoma cells Alexander) with the respective IC_50_ at 0.135, 0.033, and 0.244 μM, better than the positive control doxorubicin (0.706–2.16 μM) [76].

The fermentation broth of the marine *Streptomyces* sp. M268 contained kiamycin (**117**), possessing a 1,12-epoxybenz[a]anthracene ring system. Compound **117** showed inhibitory activities against the human cell lines HL-60, A549, and BEL-7402, with respective inhibition rates of 68.2%, 55.9%, and 31.7% at 100 µM [77]. *Micromonospora* sp., which was isolated from sediment collected off the CátBà peninsula in the East Sea of Vietnam, produced dehydrorabelomycin (**80**), phenanthroviridone (**11**), and WS-5995 A (**118**). Compound **80** showed inhibition against Kuramochi (ovarian cancer cells) with the IC_50_ at 6.72 μM, and **11** could inhibit Kuramochi and high-grade ovarian cancer cells (OVCAR4) with the respective IC_50_s of 1.11 and 4.82 μM. Compounds **11**, **80**, and **118** could inhibit murine ovarian surface epithelial (MOSE) and murine oviductal epithelial (MOE) cells with the LC_50_ of 2.85–9.80 μM, while **118** displayed cytotoxicity against L-1210 cells with the IC_50_ value about 0.5 μM [78]. The secondary metabolites of *Streptomyces* sp. SS13I contained gephyromycin C (**119**), which exhibited cytotoxicities against PC3 (prostate cancer cells, IC_50_ = 1.3 μM) and H1975 (lung adenocarcinoma cells, inhibition rate = 48% at 5 μM) [79]. A sediment-derived *Streptomyces* sp. HN-A124 (Hainan province, China) produced cysrabelomycin (**120**), which showed inhibitory activity against A2780 cells with the IC_50_ at 10.23 μM [80]. Vineomycin E (**121**), together with moromycin B (**85**) and saquayamycins B1 (**87**) and B (**37**), were generated by the marine-derived *Streptomyces* sp. OC1610.4, and all these compounds displayed potent anti-proliferation against MCF-7, MDA-MB-231, and BT-474 (breast cancer cells), with the IC_50_ values ranging from 0.16 to 7.72 µM. Meanwhile, saquayamycin B (**37**) inhibited the migration and invasion of MDA-MB-231 cells in a dose-dependent manner [81]. Moromycin B (**85**) and saquayamycins B1 (**87**) and B (**37**) were also isolated from the secondary metabolites of another marine *Streptomyces* sp. and exhibited cytotoxicity against SW480 (colon cancer cells), SW620, LoVo (colon cancer cells), HT-29, and QSG-7701 (normal hepatocyte cells), with IC_50_ values of 0.18–1.57 µM, which were comparable to or better than the positive control doxorubicin. Saq B1(**87**) could not only induce apoptosis but also inhibit invasion and metastasis in CRC (colon cancer cells) through the PI3K/AKT signaling pathway [82].

*Streptomyces* sp. XS-16 was obtained from a marine sediment sample (Naozhou Island, China) and generated compound **122**, which showed growth inhibitory activities against MDA-MB-231, K562, ASPC-1 (pancreatic cancer cells), H69AR (Adriamycin-resistant small-cell lung cancer cells), and H69 (small-cell lung cancer cells) with IC_50_ values at 0.32–5.33 µM [83]. Kumemicinones A (**123**), B (**124**), and E–G (**125**–**127**), as well as SF2315B (**128**), were isolated from the *Actinomadura* sp. KD439, associated with marine suspended matter near the coast of Kumejima Island (612 m deep, Okinawa, Japan), and all of them could inhibit P388 cells, with the IC_50_ values ranging from 1.7 to 10.7 μM [84]. Rearranged angucyclinones donghaecyclinones B (**129**) and C (**130**) were isolated from the marine sediment-derived *Streptomyces* sp. SUD119 (volcanic island, Korea). Compound **129** could inhibit hepatocellular carcinoma (SK-HEP1) cells, and **130** could inhibit HCT-116, MDA-MB-231, SNU638 (gastric cancer cells), A549, and SK-HEP1 cells, with IC_50_ values ranging from 6.0 to 9.6 μM [85]. The *Streptomyces* sp. HDN15129 isolated from a sediment sample collected in the South China Sea produced monacycliones I (**131**) and J (**132**), both of which showed inhibition against multiple human cancer cell lines such as HL-60, K562, SH-SY5Y (neuroblastoma), BEL-7402, U87 (glioblastoma), ASPC-1, and HCT-116 cells, with the IC_50_ values ranging from 3.5 to 10 μM [86].

The screening of marine actinomycete extracts against the pseudomyxoma peritonei (PMP) cell line ABX023-1 led to the isolation of grincamycins R–T (**133**–**135**) from *Streptomyces* sp. CNZ-748. Compounds **133**–**135** showed inhibitory activities against PMP501-1 and PMP457-2 cells, with IC_50_ values of 1.9–7.9 μM, and could inhibit ABX023-1 and C09-1, with IC_50_ values of 1.4–10 μM (5-fluorouracil, 2.0–4.0 μM) [87]. Caribbean sponges-derived *Streptomyces* sp. M7_15 was the producer of frigocyclinone (**136**) and monacyclinone F (**137**), which exhibited cytotoxicity against SJCRH30 (rhabdomyosarcoma cells) with the respective EC_50_ (median effect concentration) values at 5.2 μM and 0.73 μM. The result suggested that additional amino deoxy sugar subunits may be important for the activity of this class of molecules [88]. A gut-derived (*Oxya chinensis*) *Amycolatopsis* sp. HCa1 generated (2*R*, 3*R*)-2-hydroxy-5-*O*-methyltetrangomycin (**138**) together with tetrangomycin (**92**), PD116779 (**139**), and sakyomicin A–C (**140**–**142**), which displayed cytotoxicities against HeLa cells with IC_50_ values ranging from 0.11 to 0.59 µM. Compound **142** could inhibit SGC-7901 (gastric adenocarcinoma cells) with the IC_50_ of 4.41 µM, while **140** could inhibit SPC-A-1 (lung cancer cells) with IC_50_ at 8.34 µM [89].

#### 2.2.2. Terrestrial-Derived Angucyclines/Angucyclinones

**Figure 7:**
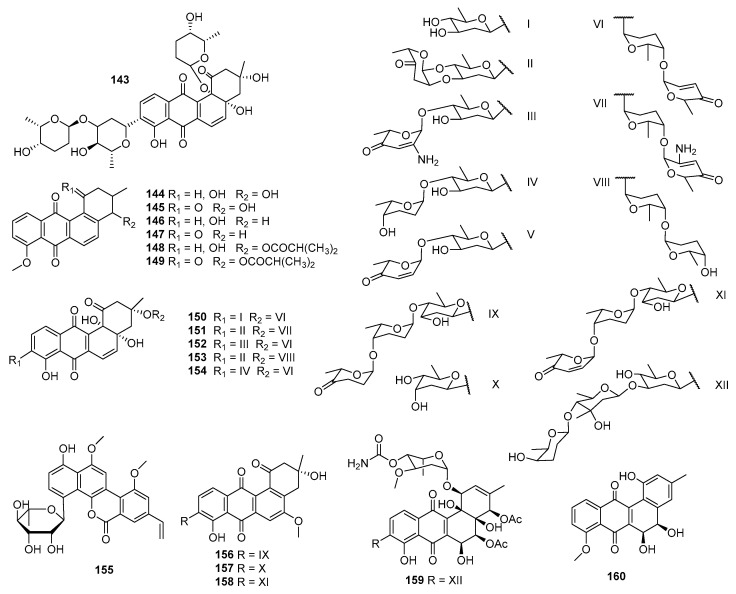
Structures of compounds **143**–**160.**

OM-4842 (**143**) was isolated from a soil-derived *Streptomyces* sp. Om-4842 (Chiba, Japan) and displayed inhibition toward doxorubicin-resistant cells of P388 at 1.5 μg/mL [90] (Figure 7). Rubiginones A1 (**144**), A2 (**145**), B1 (**146**), B2 (**147**), C1 (**148**), and C2 (**149**), secondary metabolites of the soil-derived *S. griseorubiginosus* No. Q144-2 (Andhra Pradesh, India), displayed significant potentiated cytotoxicities against vincristine-resistant P388 cells, with IC_50_ values ranging within 0.007–0.23 μg/mL [91]. KY002 and KY40-1 were both soil-derived *Streptomyces* sp. discovered in the Appalachian Mountains, USA. *Streptomyces* sp. KY002 produced moromycin B (**85**), which exhibited inhibitory activities against H-460 and MCF-7 cell lines with GI_50_s of 5.6 and 5.6 µM, respectively [92]. *Streptomyces* sp. KY40-1 generated saquayamycins G–K (**150**–**154**) as well as the known compounds saquayamycins B1(**87)**, A (**36**), and B (**37**), which displayed significant cytotoxicities against PC3 cells (IC_50_ values = 0.0075–1.759 μM) and moderate activities against H-460 cells (IC_50_ values = 3.30–7.28 μM) [93]. Polycarcin V (**155**) was produced by *S. polyformus* sp. nov. YIM 33176, which was associated with a soil sample of Vietnam, and it revealed inhibitory activities against 37 different human tumor cell lines representing 14 different solid tumor types, with the IC_70_ values ranging from 0.3 to 431.0 ng/mL, indicating a pronounced antitumor specificity [94].

The fermentation broth of *Streptomyces* sp. N05WA963 contained N05WA963 A (**156**), B (**157**), and D (**158**), which exhibited cytotoxicities against SW620, YES-4 (esophageal cancer cells), U251SP (glioma cells), K562, MDA-MB-231, and T-98 (glioma cells) with IC_50_ values of 1.0–10.3 µM [95]. Alkaline soil-derived *Streptomyces* Acta 2930 (Northumberland, UK) generated warkmycin A (**159**), with antiproliferative activities against NIH-3T3, HepG2, and HT-29 cells; the IC_50_ values were 2.74, 1.26, and 1.61 µM, respectively [96]. The Himalayan-based *Streptomyces* sp. PU-MM59 was the producer of himalaquinone G (**160**), which exhibited cytotoxicities against the PC3 and A549 cell lines with IC_50_ values of 0.32 and 1.88 μM, respectively [97]. Vineomycin A1 (P-1894B, **114**), a noncompetitive prolyl hydroxylase inhibitor (2.2 × 10^−6^ M, 50%), was isolated from the secondary metabolites of a soil-derived *S. albogriseolus* subsp. No. 1894 and was necessary for collagen biosynthesis [98]. Compound **114** showed a significant inhibitory effect on Jurkat T-cell proliferation with an IC_50_ at 0.011 μM [13]. The first total synthesis of vineomycin A1 (**114**) was accomplished in 2019, and its cytotoxicities were evaluated by MTT assay against A549, HCT-116, and Capan-1 (pancreatic cancer cells), with the IC_50_ values ranging from 0.01 to 0.64 μM. The test indicated that vineomycin A1 (**114**) effectively induced cancer cell death via apoptosis, not by acting as a DNA intercalating agent [99].

#### 2.2.3. Angucyclines/Angucyclinones from Other Sources

**Figure 8:**
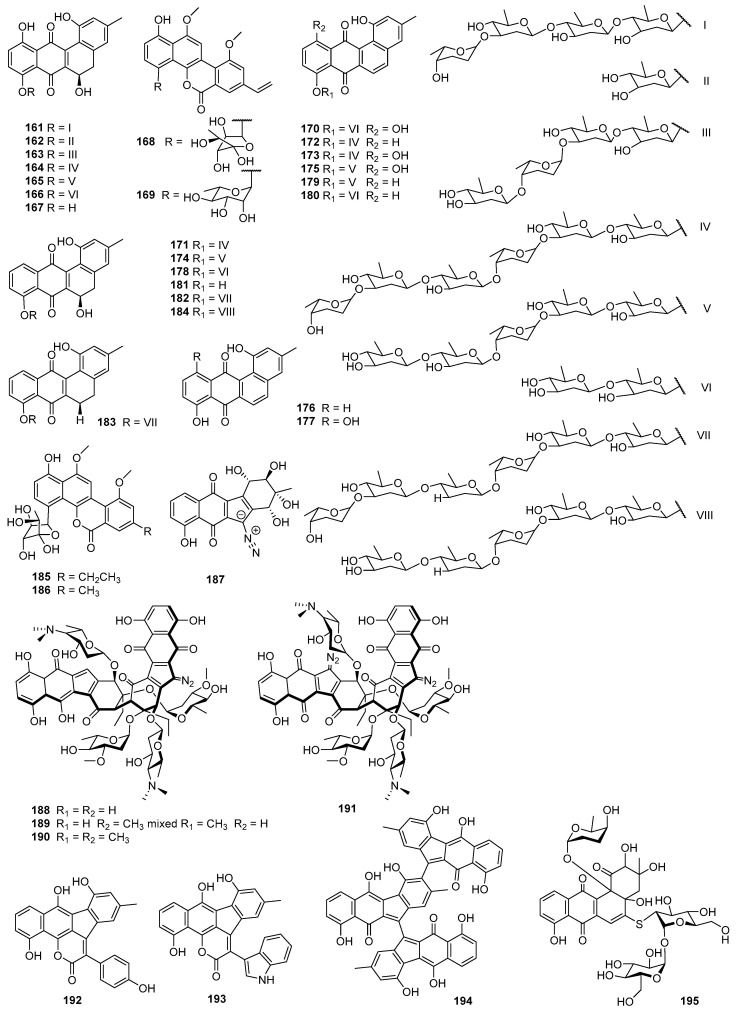
Structures of compounds **161**–**195.**

Landomycin E (**161**) was produced by *S. globisporus* 1912 [100], and it displayed inhibitory activity toward tumor cell lines via induction of apoptosis in the low micromolar range for MDA-MB-231 (IC_50_ = 0.76 mg/mL), HL-60 (1.87 mg/mL), and KB-3-1 (4.3 mg/mL) [101] (Figure 8). Landomycins I (**162**) and J (**163**), together with landomycins A (**164**), B (**165**), E (**161**), and D (**166**), and one landomycinone (**167**) [102,103,104,105,106,107], were generated by a mutant strain of *S. cyanogenus* whose glycosyltransferase encoded by *lanGT3* was over-expressed [108]. Complementation of gilvocarcin for the mutant *S. lividans* TK24 (cosG9B3-U), in which the biosynthesis of the natural sugar donor substrate was compromised with various deoxy sugar plasmids, led to the production of the gilvocarcin analogues gilvocarcin V (**5**), 4′-OH-gilvocarcin V (**168**), D-olivosyl-gilvocarcin V (**169**), and polycarcin V (**155**) with altered saccharide moieties [109]**.** Compounds that differed in their sugar moieties showed inhibition against LL/2 (mouse lung cancer), MCF-7, and NCI-H460 cell lines, indicating that the anticancer activity of landomycins did not increase simultaneously with the elongation of their oligosaccharide chain lengths [108,109]. However, other studies showed different results in the structure–activity relationship of landomycins.

Landomycins R–W (**170**–**175**) along with tetrangulol (**176**) [110]; 5,6-anhydrolandomycinone (**177**) [102]; landomycinone (**167**); landomycins A (**164**), B (**165**), D (**166**), F (**178**) [111], M (**179**) [112], and O (**180**) [113]; and tetrangomycin (**92**) were isolated from the culture broth of *S. cyanogenus* S-136 [114]. 11-Deoxylandomycinone (**181**) and landomycins X–Z (**182**–**184**) were produced by the mutant strain of *S. cyanogenus* K62 [115]. These compounds showed varying degrees of cytotoxic activity toward MCF-7 (estrogen-sensitive) and MDA-MB-231 (estrogen-insensitive) cell lines. Compounds **164**, **167**, and **177** showed the best combined activities to both MCF-7 and MDA-MB-231 cells, with **177** for the former and **167** and **164** for the latter. Compounds **173**–**175** and **181**–**184** showed activities against MCF-7, with IC_50_ values of 1.0–6.7 µM, while compounds **172**–**175** and **181**–**184** could inhibit MDA-MB-231, with IC_50_ values of 1.2–2.5 µM. Compounds with shorter saccharidal moieties were less potent against MCF-7. The fact that most landomycins with bioactivities had either long penta- or hexasaccharide chains or no sugars at all suggests that the large molecules may act by a different mode of action compared with their small sugar-free congeners [114,115].

The fermentation broth of *Streptomyces* contained gilvocarcin V (**5**), which displayed cytotoxicities against sarcoma 180, Ehrlich carcinoma, Meth I fibrosarcoma, MH134 (mouse hepatoma), and P388 cells. After intraperitoneal administration of gilvocarcin V (**5**) to mice bearing Ehrlich ascites carcinoma, 40% of the treated mice survived for 60 days [31]. The inactivation of the *gilU* gene in the mutant *S. lividans* TK24 (cosG9B3-U) led to the production of three analogues of the gilvocarcin-type aryl-C-glycoside compounds, 4′-hydroxy gilvocarcins E (**185**), M (**186**), and V (**168**), which showed different degrees of activity in the anticancer assay. The activity of **185**, which lacks an essential vinyl residue for DNA binding, was lower than those of **168** and **186**. Nevertheless, the introduction of the 4′-OH group changed an inactive gilvocarcin E (**161**) into a moderately active one (**185**) [109,116]. Kinamycin F (**187**), as a secondary metabolite of *S. murayamaensis*, was found to induce apoptosis and downregulate cyclin D3 in K562 cells, and it induced single-stranded DNA breaks and inhibited the activity of topoisomerase IIα with an IC_50_ of 0.33 μM [69,117]. *Salinispora pacifica* DPJ-0019 (NRRL 50168), which was acquired from the USDA Agricultural Research Service, generated (−)-lomaiviticins C–E (**188**–**190**) as well as lomaiviticin A (**191**) and kinamycin C (**102**), all of which displayed significant cytotoxicities against K562, LNCaP (prostate cancer), HCT-116, and HeLa cells, with the IC_50_ values ranging from 2 to 589 nM [118]. Inactivation of the flavoenzyme-encoding gene of *lsO*1 in fluostatin biosynthesis led to the isolation of fluostarenes A (**192**), B (**193**), and PK1 (**194**). Fluostarene B (**193**) was cytotoxic toward SF-268, MCF-7, HepG2, and A549 cell lines, with IC_50_ values at 7–10μM, not as good as Adriamycin (1.13–1.42 μM) [119]. BE-7585A (**195**), which is characterized by a 2-thiosugarand, was isolated from a culture broth of *Amycolatopsis orientalis* subsp. vinearia and exerted cell inhibitory effects against mouse Ehrlich ascites carcinoma, with an IC_50_ of 8.0 μg/mL. The antitumor mechanism of **195** might be based on the inhibition toward thymic acid synthase, one of the key enzymes of nucleic acids [120].

### 2.3. Antibacterial or Antifungal Activities

#### 2.3.1. Marine-Derived Angucyclines/Angucyclinones

**Figure 9:**
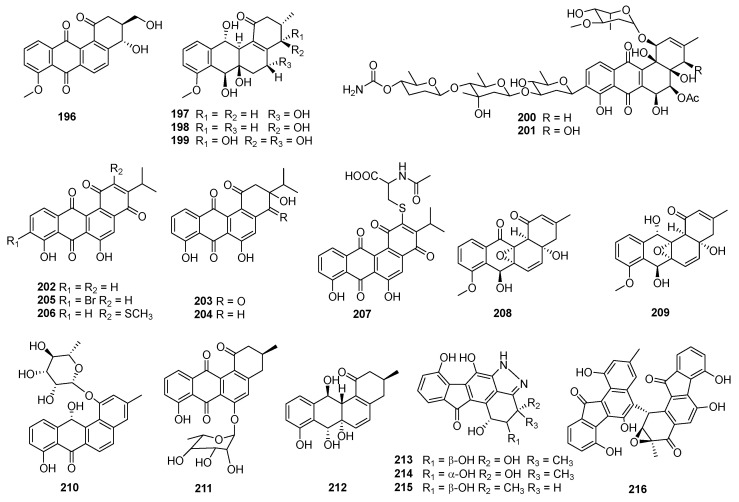
Structures of compounds **196**–**216.**

Fujianmycin C (**196**) was isolated from the fermentation broth of the marine actinomycetes *Streptomyces* sp. B6219, which was isolated from the sediment of the Galapagos mangrove (Figure 9). Fujianmycin C (**196**) showed weak antibacterial activity against *S. viridochromogenes* Tṻ57, with an inhibition zone of 14 mm at 40 μg/tablet [121]. *Saccharothrix espanaensis* AN113 was isolated from the marine mollusk *Anadara broughtoni* and yielded three antibiotics, saccharothrixins A–C (**197**–**199**), which displayed moderate activities against *B. subtilis*, *E. faecium*, and *Xanthomonas* sp. pv. Badrii at 100 μg/mL [122]. In the process of *S. pratensis* NA-ZhouS1’s culture, the addition of 100 µM nickel ion led to the production of antibacterial gypenocyclins stremycins A (**200**) and B (**201**), both of which could inhibit *P. aeruginosa* CMCC (B) 10104, MRSA, *K. pneumonia* CMCC (B) 46117, and *E. coli* CMCC (B) 44102 with equal MIC values of 16 µg/mL, and they showed inhibition against *B. subtilis* CMCC (B) 63501, with MIC values of 8–16 µg/mL. This is the first report that a new angucycline compound has been discovered through a “metal stress technique” [123]. The *Nocardiopsis* sp. HB-J378 is associated with a marine sponge *Theonella* sp. and produced nocardiopsistins A–C (**202**–**204**), which displayed activities against MRSA with MICs of 3.12–12.5 μg/mL. Among them, the MIC of nocardiopsistin B (**203**) was comparable to chloramphenicol (positive control, 3.12 μg/mL) [124]. The brominated nocardiopsistin D (**205**) and sulfur-containing nocardiopsistins E (**206**) and F (**207**) were also identified from *Nocardiopsis* sp. HB-J378; all of them showed anti-MRSA activities, with MICs at 0.098, 3.125, and 0.195 μg/mL, respectively. The single bromination in **205** drastically enhanced the anti-MRSA activity by 128-fold, and it acquired activities against vancomycin-resistant *S. aureus* (VRSA), *E. faecium*, and *B. cereus* [125].

*S. lusitanus* OUCT16-27, isolated from deep-sea sediment (4495 m deep, the Indian Ocean), produced the antibiotics grincamycins L (**82**) and I (**116**), and both of them displayed bioactivities against *E. faecium*, *E. faecalis*, and *S. aureus*, with MICs of 3.12–6.25 μg/mL [126]. A type II PKS gene cluster harboring genes to encode several distinct oxidoreductases were identified from a rare marine actinomycete *Saccharothrix* sp. D09 by genome mining. The study of the gene cluster led to the isolation of the angucycline derivatives **208**–**210**, all of which showed bioactivities toward *Helicobacter pylori* with MIC values ranging from 16 to 32 μg/mL [127]. The same research group’s study of sediment-derived *Streptomyces* sp. BHB-032 (Bohai Gulf, China) led to the discovery of atramycin C (**211**), bearing an *O*-6 rhamnose side chain, and a highly hydroxylated angucyclinone emycin G (**212**). Compounds **211** and **212** exhibited moderate activities toward *S. aureus* CMCC 26003, *Nocardia*, *B. cereus* CMCC 32210, and *B. subtilis* CMCC 63501, with MICs of 16–64 μg/mL, but not as active as the positive control (ampicillin, MIC < 1 μg/mL) [128]. Study of *M. rosaria* SCSIO N160 led to the discovery of pyrazolofluostatins A–C (**213**–**215**), which possess a benzo[cd]indeno[2,1-f]indazol skeleton with a pyrazole-fused 6/5/6/6/5 pentacyclic ring system. Compounds **213**–**215** showed weak bioactivities against pathogens including *E. coli* ATCC 25922, *S. aureus* ATCC 29213, *B. thuringensis* SCSIO BT01, *B. subtilis* SCSIO BS01, and *C. albicans* ATCC 10231. Pyrazolofluostatin A (**213**) also exhibited moderate antioxidant activity (EC_50_ = 48.6 μM) [129]. Further, the expression of the fluostatin structural genes of *M. rosaria* SCSIO N160 in a heterologous host, *S. coelicolor* YF11, led to the isolation of an unusual heterodimer difluostatin A (**216**). Compound **216** exhibited antibacterial activities against *K. pneumoniae* ATCC 13883, *Aeromonas hydrophila* ATCC 7966, and *S. aureus* ATCC 29213, with respective MICs of 4, 4, and 8 µg/mL, while the MIC values of the positive control trimethoprim (TMP) were 0.25, 0.25, and 4 µg/mL, respectively [130].

#### 2.3.2. Terrestrial-Derived Angucyclines/Angucyclinones

**Figure 10:**
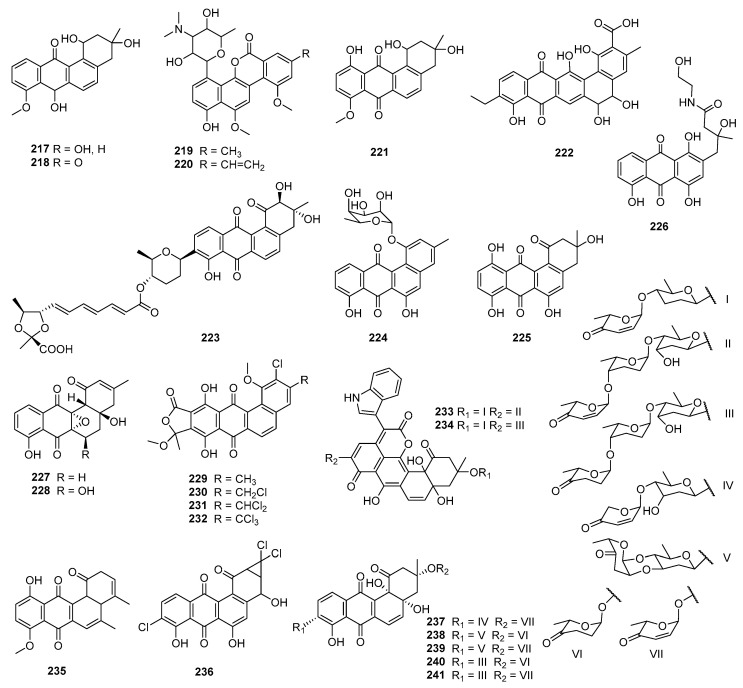
Structures of compounds **217**–**241.**

Sakyomicins A–C (**140**–**142**), which were isolated from the fermentation extract of soil-derived actinomycete *Nocardia.* sp. M-53, displayed selective inhibitory activities against several Gram-positive bacteria (*Bacillus*, *Staphylococcus*, *Micrococcus*, *Cotrnebacterium*, *Mycobacteriu*), with MIC values ranging from 0.78 to 12.5 μg/mL [131]. The research on soil-derived *Streptomyces* sp. DSM 4769 (Adamata, India) led to the discovery of the antibiotics SM 196 A (**217**) and B (**218**), both of which could inhibit *S. aureus* H 503 (MIC = 100 and 25 μg/mL, respectively), *S. pyogenes* (MIC = 12.5–25 and 6.25 μg/mL, respectively) [132] (Figure 10). *Streptomyces* sp. WK-6326, which was isolated from a soil sample collected in Utah, USA, produced deacetylravidomycin M (**219**) and deacetylravidomycin (**220**). Compound **219** could inhibit the growth of *B. subtillis* and *M. luteus* (MIC = 25 μM/mL), and **220** displayed antimicrobial activities against the Gram-positive bacteria *B. subtillis*, *S. aureus*, *M. luteus*, and *M. smegmatis*, with MICs ranging from 3.0 to 5.0 μM/mL. Meanwhile, **219** inhibited IL-4-induced CD23 expression in U937 cells but had no cytotoxic effect, while **220** was identified as an interleukin-4 (IL-4) signal transduction inhibitor [133]. Seitomycin (**221**) and tetrangulol methyl ether (**68**) were isolated from the fermentation extracts of two terrestrial *Streptomyces* spp., GW19/1251 and GW10/1118. Compounds **221** and **68** exhibited moderate antibacterial activities in the agar diffusion assay toward *B. subtilis*, *S. viridochromogenes* Tü57, *S. aureus*, and *E. coli*, with the inhibition zones of 8–29 and 17–20 mm at 5 μg/disk, respectively. Compound **221** also showed weak phytotoxicity against *Chlorella vulgaris* and *C. sorokiniana* [134].

*Streptosporangium* sp. Sg3, a soil-derived actinomycete from Algeria, produced angucyclinone R2 (**222**), with antimicrobial activity [135]. Compound **222** significantly inhibited *M. luteus* ATCC 9314 and *B. subtilis* ATCC 6633, with MICs of 0.5 and 1.0 μg/mL, and it could also moderately inhibit *S. aureus* CIP 7625, *Listeria monocytogenes* CIP 82110, and *M. smegmatis* ATCC 607, with MICs of 10, 40, and 50 μg/mL, respectively [136]. Waldiomycin (**223**) was isolated from the strain MK844-mF10 which was associated with soil collected at Shiogama, Miyagi, Japan. Waldiomycin (**223**) exhibited activities against *S. aureus* and *B. subtilis*, with IC_50_ values at 8.8 and 10.2 μM, respectively, and could also inhibit the methicillin-resistant ones, with MICs ranging from 4 to 8 μg/mL [137]. Studies on the antibacterial mechanism of waldiomycin (**223**) showed that it targeted WalK histidine kinases and inhibited the WalR regulon genes expression, thereby affecting both cell wall metabolism and cell division [138]. An angucycline containing *O*-glycosylated 6-deoxy-a-L-talose, amycomycin D (**224**), produced by *Kitasatospora* sp., displayed inhibition toward *S. aureus* Newman, *Pichia anomala*, *Mucor hiemalis*, and *E. coli* ToIC, with MICs of 9.21–14.6 μM [139]. The expression of the landomycin A structural genes LanI and LanK in a heterologous host, *S. albus* J1074, led to the isolation of 6,11-dihydroxytetrangulol (**93**), 11-hydroxyrabelomycin (**225**), and fridamycin G (**226**). Compounds **93** and **225** exhibited activities against *B. subtilis* DSM 1092 and *M. luteus* DSM 20030, while **226** could inhibit *S. aureus* Newman; all the MICs were 1 µg/mL [140]. *Streptomyces* sp. KMC004, which was associated with acid wastewater collected from coal mines (Yeongdong, Gangneung, Republic of Korea), produced angumycinones A (**227**) and B (**228**), and both compounds showed comparable inhibitory activities against *M. luteus*, *E. hirae*, and MRSA with ampicillin (MICs = 0.78–12.5 μg/mL); the MIC values ranged from 0.78 to 12.5 μg/mL [141]. *Actinoallomurus* sp. ID145698 was the producer of angucyclinone allocyclinones A–D (**229**–**232**), which contained chlorine atom substitutions. Compounds **229**–**232** exhibited antibacterial activities against *S. aureus* ATCC 6538P, *S. pyogenes* L49, *E. faecalis* L560, and *E. faecium* L569; the MIC values ranged from 0.25 to 4 μg/mL, and the antibacterial activity increased as the number of chlorine atoms increased [142].

A Saharan soil-derived *actinobacterium* PAL114 (Mzab region, southern Algeria) produced mzabimycins A (**233**) and B (**234**), which contained L-tryptophan and glucoside derivatized chromophore on account of the addition of L-tryptophan in the fermentation; both of them exhibited antibacterial activities against *M. flavus* ATCC 9314 and *L. monocytogenes* ATCC 13932, with MIC values ranging from 15 to 40 μg/mL [143]. Based on a bioassay-guided isolation, **235** was discovered from the stem bark extracts of *Stereospermum fimbriatum* and exhibited bioactivities against *S. epidermidis* ATCC 12228, MRSA, and *S. aureus* ATCC 25923, with MIC values of 3.13–6.25 μg/mL [144]. Actinomycetes strain RI104-LiC106, associated with lichen, generated a 1,1-dichlorocyclopropane-containing angucycline, JBIR-88 (**236**), which exhibited antibacterial activity against *M. luteus* when the paper contained 25 μg of the compound (inhibition zone, 11mm) [145]. A soil-derived *Streptomyces* sp. TK08046 (Shizuoka, Japan) was the producer of saprolmycins A–E (**237**–**241**), which displayed inhibition against *Saprolegnia parasitica,* with respective MICs of 0.0039, 8, 1, 1, and 0.0078 µg/mL. Compound **241** could also inhibit *S. aureus*, *B. subtilis*, and *Daphnia pulex,* with MICs at 15.6, 7.8, and 4.5 µg/mL, respectively [146]. *S. griseus* NTK 97 was isolated from a terrestrial sample of Terra Nova Bay at Edmondson Point, Antarctica and was the producer of frigocyclinone (**136**), which could inhibit *B. subtilis* DSM 10 and *S. aureus* DSM 20231, with MICs of 10 and 33 μM, respectively (positive control vancomycin and erythromycin, MICs = 1 μM) [147].

#### 2.3.3. Angucyclines/Angucyclinones from Other Sources

**Figure 11:**
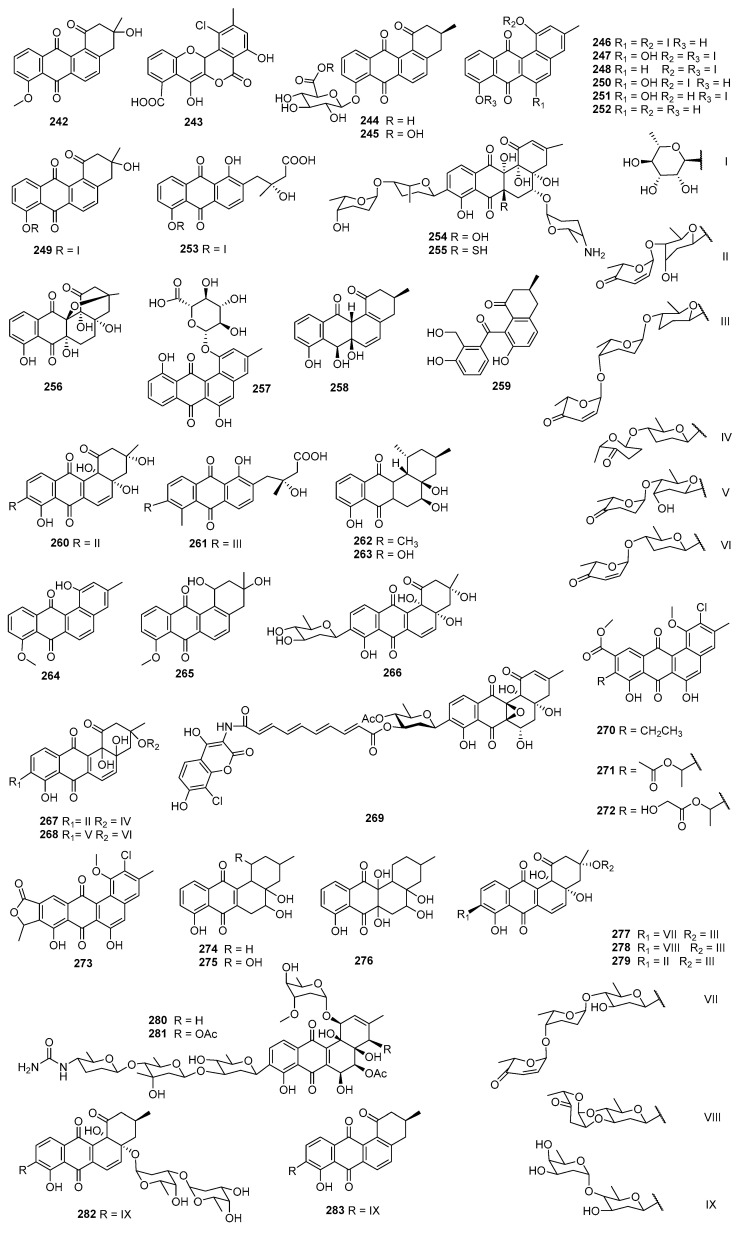
Structures of compounds **242**–**283.**

6-Deoxy-8-*O*-methylrabelomycin I (**242**), produced by *S. tsusimaensis* MI310-38F7, showed inhibitory activities against various Gram-positive bacteria (*S. aureus* Smith, multi-resistant *S. aureus* MS9610, *M. luteus* PCI 1001, and *B. subtilis* NRRLB-558), with MIC values between 12.5 and 25.0 μg/mL, but it displayed no activity against Gram-negative bacteria [148] (Figure 11). Ecological cultivation of *Actinomadura* sp. RB29 and mass spectral-mediated molecular network analysis led to the expression of a silent gene cluster and the discovery of maduralactomycin A (**243**), which exhibited moderate activities against VRE (few colonies in the inhibition zone, 13 mm) and *M. vaccae* (12 mm) using the broth dilution method [149]. Acidonemycins A (**244**) and B (**245**) were discovered from the acidic culture (pH 5.4) of *S. indonesiensis* DSM 41759 and exhibited in vitro antivirulence activities against MRSA. Both compounds could inhibit the production of PSM and the formation of biofilm but not a significant growth inhibition. Further study indicated that the PSM and biofilm inhibitory activities of **244** and **245** were due to the (+)-ochromycinone aglycone moiety [150].

### 2.4. Other Bioactivities

#### 2.4.1. Marine-Derived Angucyclines/Angucyclinones

The inhibition of dopamine *S*-hydroxylase caused by **1** was examined according to the method of NAGATSU, and the inhibition percentage at 0.1 μg/mL was 65.2% [16]. *Actinokineospora spheciospongiae* EG49 was isolated from the Red Sea sponge *Spheciospongia vagabunda*, and the fermentation broth contained actinosporins A (**246**) [151], C (**247**), and D (**248**) [152]. Actinosporin A (**246**) exhibited selective inhibitory activity against *Trypanosoma brucei brucei* with an IC_50_ value of 15 µM. The antioxidant potential of actinosporins C (**247**) and D (**248**) was demonstrated using the FRAP assay. Meanwhile, at 1.25 µM, actinosporins C (**247**) and D (**248**) showed significant antioxidant and protective capacity against the genomic damage induced by hydrogen peroxide in the HL-60 cell line. Furthermore, co-cultivation of *Actinokineospora* sp. EG49 with *Rhodococcus* sp. UR59 and antimalarial-guide isolation led to the discovery of actinosporins E (**249**), H (**250**), and G (**251**), and tetragulol (**252**), which exhibited antimalarial activities and good binding affinity to lysyl-*t*RNA synthetase (PfKRS1), with IC_50_ values of 9–13.5 μg/mL [153]. Solid cultivation of *Actinokineospora* sp. led to the generation of fridamycin H (**253**), which exhibited growth inhibition toward *T. brucei* TC221; the IC_50_ values after 48 h and 72 h were 7.18 and 3.35 μM, respectively [154].

#### 2.4.2. Terrestrial-Derived Angucyclines/Angucyclinones

Highly oxygenated grecocycline D (**254**) was obtained from the extract of soil-derived *Streptomyces* sp. KCB15JA014 (Jeju Island, Republic of Korea), and showed a 46.2% inhibition rate at 50 μM against the IDO (indoleamine 2,3-dioxygenase) enzyme [155]. *Streptomycete Acta* 1362, which was isolated from pine rhizosphere soil on Crete, was the producer of grecocycline B (**255**), inhibiting protein tyrosine phosphatase 1B (PTP1B), with an IC_50_ at 0.52±0.17 μM [156]. Highly oxygenated gephyromycin (**256**) was isolated from the fermentation broth of Antarctic soil-derived *S. griseus* and demonstrated glutaminergic agonistic properties. When **256** was incubated with neurons for 5 min at 3 mg/mL, the concentration of intracellular Ca^2+^ increased twofold [157].

*Streptomyces* sp. KCB15JA151 isolated from soil samples collected in Jeju Island, Republic of Korea, produced pseudonocardone D (**257**). Compound **257** could inhibit cell proliferation induced by 17β-estradiol, which suggested that **257** might be an ER-α (estrogen receptor) antagonist [158]. The research on a soil-derived *Streptomyces* sp. KIB-M10 led to the isolation of cangumycins B (**258**) and E (**259**), which exhibited potent immunosuppressive activities (IC_50_ values = 8.1 and 2.7 μM, respectively) against human T-cell proliferation at a non-cytotoxic concentration. [159]. Fermentation of soil-derived *Streptomyces* sp. #AM1699 (Queensland, Australia) led to the isolation of saquayamycin A1 (**260**) and A-7884 (**34**) and vineomycin C (**261**), which showed inhibitory activities in the inducible nitric oxide synthase (iNOS) assay with IC_50_ values of 101.2, 43.5, and 25.3 μM, respectively, comparable to the known standard inhibitors N^G^-monomethyl-L-arginine (L-NMMA, 17.0μM) and N^G^-nitro-L-arginine (L-NNA, 71.0 μM) [160]. Compounds **262**, **263**, and **147** exhibited antimalarial activities against *P. falciparum* K1, with IC_50_ values of 3.9–6.0 µM [161]. The solid-state fermentation of soil-derived *Streptomyces* sp. P294 led to the isolation of X-14881 E (**264**) [162], which could inhibit *P. burgneri hepatis* with an IC_50_ at 3 μM [36]. OM-4842 (**143**) showed an inhibitory effect on platelet aggregation induced by ADP (adenosine diphosphate), arachidonic acid, PAF (platelet-activating factor), or collage; the MICs were 5.0, 12.5, and 25 μg/mL, respectively, better than kerriamycins B (**44**) and C (**45**), produced by *S. violaceolatus* [36,90]. Compound **265** showed a significant inhibitory activity against the DNA viruses Herpes simplex I and II, with MIC values of 0.55 and 4.54 μg/mL, respectively [132].

#### 2.4.3. Angucyclines/Angucyclinones from Other Sources

Aquayamycin **266** is a noncompetitive inhibitor of tyrosine hydroxylase and dopamine β-hydroxylase, and it can inhibit the activity of enzymes by 50% with Ki values of 0.36 μM, 0.21 μM at concentrations of 0.37 μM, 0.40 μM, respectively. The inhibition of **266** was not affected by cofactors such as ascorbic acid and fumarate, and the inhibitory mechanism was possibly due to the chelating action of **266** on protein-bound copper. However, the inhibition could be reversed by the addition of Fe^2+^ but not Cu^2+^ [163,164]. Meanwhile, **266** also showed noncompetitive inhibition against tryptophan 5-mono-oxygenase (1.0 × 10^−7^ μM, 40%) [165]. Saquayamycins E (**267**) and F (**268**), produced by actinomyces MK290-AF1, were reported to inhibit the FPTase (farnesyl protein transferase) from bovine brains, with IC_50_ values of 1.8 and 2.0 μM, respectively, and they had a noncompetitive inhibitory effect on this enzyme [166]. The heterogeneous expression of the biosynthetic gene cluster from simocyclinones in *S. coelicolor* YF11 M1152, and the deletion of the keto-reducing gene *simC7,* related to angucyclinone, led to the production of 7-oxo-simocyclinone D8 (**269**), while simocyclinone D8 (**42**) was produced by *S. antibioticus* Tü 6040. Both compounds displayed inhibitory activities against DNA gyrase with the IC_50_ values of 50 μM and 0.1–0.6 μM, respectively. The production of **269** was related to the absence of simC7, indicating that SimC7 catalyzed the conversion of **269** to **42** as an NAD(P) (nicotinamide adenine dinucleotide phosphate) H-dependent ketoreductase, and the reduction of the carbonyl group by *SimC7* was essential for the compound to bind to the enzyme with high affinity [167]. The mycelium extract of *Streptomyces* sp. DSM 17045 contained the PPAR-γ (proliferator-activated receptor gamma) antagonists chlorocyclinones A–D (**270**–**273**). When using an AlphaScreen assay, which was able to displace rosiglitazone from the PPAR-γ ligand-binding domain (LBD) in a scintillation proximity assay (SPA), **270**–**273** antagonized rosiglitazone-induced peroxisome PPAR-γ activation with an IC_50_ < 0.4 µM in vitro. The compounds were also proved to be active in a cell-based reporter gene assay, antagonizing rosiglitazone-induced PPAR-γ activities with IC_50_ values between 0.60 and 7.0 µM [168].

High-throughput screening of microbial metabolites led to the discovery of the IDO1 inhibitors **274**–**276,** which showed inhibition of the production of kynurenine, with respective IC_50_ values at 0.230, 0.067, and 5.88 µM. Enzyme kinetics experiments showed that compound **274** was a reversible noncompetitive inhibitor of IDO1 [169]. Fluostarenes A (**192**), B (**193**), and PK1 (**194**) showed α-glucosidase inhibitory activities, with IC_50_ values of 0.89, 1.58, and 0.13 μM, respectively (positive control acarbose, 0.015μM) [119]. The secondary metabolites of *S. matensis* A-6621 contained PI-080 (**277**), PI-083 (**96**), PI-085 (**278**), and PI-087 (**279**), with inhibitory effects on platelet aggregation in rabbits. Using ADP, collagen, and arachidonic acid as aggregating agents, the IC_50_ values ranged from 1.56 to 30.4 μg/mL [170]. P371A1 (**280**) and P371A2 (**281**), produced by *Streptomyces* sp. P371, exhibited inhibitory activities against pentagastrin-stimulated acid secretion and also showed protective activities against HCl/ethanol- and indomethacin-induced gastric lesions [171]. P371A1 (**280**) showed an inhibition rate of 61% on pentagastrin-stimulated acid secretion, suggesting the compound served as a CCK B/gastrin receptor antagonist. When administered interperitoneally at a dose of 10 mg/kg, **280** provided 83.6% inhibition against HCl/ethanol (60% ethanol in 150 mL HCl)-induced lesions, and at a dose of 25 mg/kg, **280** provided 72.8% inhibition against indomethacin-induced lesions [172]. Glycosylated streptocyclinones A (**282**) and B (**283**) were isolated from *Streptomyces* sp. and displayed antioxidant properties and modulation of the inflammatory response. Streptocyclinones A (**282**) and B (**283**) were able to protect SH-SY5Y neuroblastoma cells from H_2_O_2_-induced oxidative injury by activating the nuclear factor E2-related factor (Nrf2), and they were also able to inhibit the activity of β-secretase 1 and decrease the release of reactive oxygen species in BV2 (mouse glioma cells) stimulated with Aβ (amyloid β protein) [173]. Compound **208** displayed anti-inflammatory activity by inhibiting the production of NO with an IC_50_ at 28 μM [128]. Further research showed that **242** could also inhibit liver-stage *P. burgneri* with the IC_50_ of 18.5 μM [162].

**Table 1:** Angucyclines/angucyclinones with bioactivities.

Cytotoxic Activities			
Compound No.	Producer	Model of Bioactivities	Reference
**1**	*Chainia purpurogena*	EHRLICH ascites	[16]
**2**	*Streptomyces* sp. HB202	HepG2, HT-29, GXF251L, LXF529L, MAXF401NL, MEXF462NL, PAXF1657L, RXF486L	[17]
**5**	*Streptomyces* sp. QD01-2 *S. gilvotanarens* NRRL 11382 Mutant strain of *S. cyanogenus*	MCF-7, K562, P388 Sarcoma 180, P388, Ehrlich carcinoma, Meth I fibrosarcoma, MH134 LL/2, MCF-7, NCI-H460	[18] [31] [108,109]
**6**	*S. gilvotanarens* NRRL 11382	Sarcoma 180, P388	[31]
**7**–**9**	*S. fradiae* PTZ0025	HCT-15, SW620, C6	[19]
**10**	*Micromonospora rosaria* SCSIO N160 *Streptomyces* sp. XZHG99T *Streptomyces* sp. IB201691-2A	SF-268, MCF-7, NCI-H460 A549, H157, MCF-7, MDA-MB-231, HepG2 Huh7.5, SW620, A549	[20] [61] [62]
**11**	*M. rosaria* SCSIO N160 *Micromonospora* sp.	SF-268, MCF-7, NCI-H460 Kuramochi, OVCAR4, MOSE, MOE	[20] [78]
**12**, **14**–**16**	*M. echinospora* SCSIO 04089	SF-268, MCF-7, HepG2	[21]
**13**	*M. echinospora* SCSIO 04089	HepG2	[21]
**18**, **19**	*S. pratensis* KCB-132	LS180	[22]
(+)-**21**	*S. pratensis* KCB-132	NCI-H460	[24]
(–)-**21**	*S. pratensis* KCB-132	NCI-H460, HepG2	[24]
**22**	*S. pratensis* KCB-132	Colon 38, HeLa cells	[24]
**25**, **26**	*S. ardesiacus* 156VN-095	ACHN, HCT-15, MDA-MB-231, NCI-H23, NUGC-3, PC-3	[27]
**28**	*S. ardesiacus* 156VN-095 *Streptomyces* sp. BCC45596	ACHN, HCT-15, MDA-MB-231, NCI-H23, NUGC-3, PC-3 KB, MCF-7, NCIH187, Vero	[27] [28]
**29**–**31**	*Streptomyces* sp. BCC45596	KB, MCF-7, NCIH187, Vero	[28]
**33**	*Streptomyces* sp. SCSIO 11594	A594, CNE2, HepG2, MCF-7	[29]
**34**, **35**	*S. lusitanus* SCSIO LR32	MDA-MB-435, MDA-MB-231, NCI-H460, HCT-116, HepG2, MCF10A	[30]
**36**	*S. nodosus* MH190-16F3 *Streptomyces* sp. KY40-1	P388/S, P388/ADR, L-1210, A-549, HT-29 PC3, H-460	[32,33] [93]
**37**	*S. nodosus* MH190-16F3 *S. lusitanus* SCSIO LR32 *Streptomyces* sp. *Streptomyces* sp. OC1610.4 *Streptomyces* sp. *Streptomyces* sp. KY40-1	P388/S, P388/ADR, L-1210, A-549, HT-29 Jurkat T cells HepG-2, SMMC-7721, PLC-PRF-5 MCF-7, MDA-MB-231, BT-474, MDA-MB-231 SW480, SW620, LoVo, HT-29, QSG-7701, CRC PC3, H-460	[32,33] [13] [76] [81] [82] [93]
**38**	*Amycolatopsis* sp. HCa1	HeLa	[89]
**40**	*S. nodosus* MH190-16F3	P388/S, P388/ADR	[32]
**41**, **42**	*S. antibioticus* Tü 6040	HMO2, MCF-7	[34,35]
**43**–**46**	*S. capoamus*	M1	[36]
**47**	*S. griseoincarnatus* *S. lusitanus* SCSIO LR32	P388 B16, HepG2, SW-1990, HeLa	[38] [75]
**48**–**50**	*S. venezuelae* ISP5230	MDA-MB-435, T-47D	[39]
**59**	*Streptomyces* sp. AC113 *Streptomyces* sp. CB01913	B16, HT29 SF295, H226, M14	[44] [63]
**60, 61**	*Streptomyces* sp. AC113	B16, HT29	[44]
**64**	*Streptomyces* sp. Acta 3034	HepG2, NIH 3T3	[51]
**65**, **66**	*Saccharopolyspora* BCC 21906	KB, MCF-7, NCI-H187	[52]
**69**–**71**	*S. salbus*	HCT-116	[53]
**72**	*S. chattanoogensis* L10 (CGMCC 2644)	MCF-7	[54]
**73**	*S. chattanoogensis* L10 (CGMCC 2644)	MCF-7, HepG2	[54]
**75**	*S. blastomycetica* F4-20	BGC823, HeLa	[56]
**76**, **77**	*S. bulli* GJA1, *Gardenia jasminoides*	OV90, ES2	[57]
**78**	*Dermatophilaceae Aptenodytes* NJES-13T	HL-60, Bel-7402, A549	[58]
**80**	*Micromonospora* sp.	Kuramochi, MOSE, MOE	[78]
**82**–**84**, **86**	*Streptomyces* sp. XZHG99T	A549, H157, MCF7, MDA-MB-231, HepG2	[61]
**85**	*Streptomyces* sp. XZHG99T *Streptomyces* sp. OC1610.4 *Streptomyces* sp. *Streptomyces* sp. KY002	A549, H157, MCF7, MDA-MB-231, HepG2 MCF-7, MDA-MB-231, BT-474, MDA-MB-231 SW480, SW620, LoVo, HT-29, QSG-7701, CRC H-460 and MCF-7	[61] [81] [82] [92]
**87**	*Streptomyces* sp. XZHG99T *Streptomyces* sp. OC1610.4 *Streptomyces* sp. *Streptomyces* sp. KY40-1	A549, H157, MCF7, MDA-MB-231, HepG2 MCF-7, MDA-MB-231, BT-474, MDA-MB-231 SW480, SW620, LoVo, HT-29, QSG-7701, CRC PC3, H-460	[61] [81] [82] [93]
**88**	*Streptomyces* sp. IB201691-2A	Huh7.5, SW620	[62]
**89**	*Streptomyces* sp. IB201691-2A	Huh7.5, SW620, A549	[62]
**90**	*Streptomyces* sp. CB01913	SF295, H226	[63]
**92**	*Streptomyces* sp. CB01913	SF295, H226, M14	[63]
**93**	*S. lividans* TK23	HL-60	[64]
**94**	*Nocardia lurida*	9KB, 9PS	[65]
**95**	*N. lurida*	9KB, 9PS, 9ASK	[65,66]
**96**	*Streptomyces matensis* A-6621	KB	[67]
**97–99**	*Nocardia* sp. IFM 0089	L1210, P388, P388/ADR	[68]
**100**	*S. murayamaensis*	CHO	[69]
**102**	*S. murayamaensis* *Salinispora pacifica* DPJ-0019	CHO K562, LNCaP, HCT-116, HeLa	[69] [118]
**104**, **105**	Unknown actinomycetes	Most of the cancer cells	[71]
**106**	*S. aureofaciens* CCM 3239	A2788, A2780/CP, MDA-MB-231, MCF-7	[72]
**107**, **108**	*Streptomyces* sp. CNH990	HCT-116	[73]
**109**	*Saccharopolyspora taberi* PEM-06-F23-019B	MDA-MB-231, HT-29, A-549	[74]
**110**–**112**	*S. lusitanus* SCSIO LR32	B16, HepG2, SW-1990, HeLa	[75]
**113**, **115**	*S. lusitanus* SCSIO LR32	Jurkat T cells	[13]
**114**	*S. lusitanus* SCSIO LR32 *S. albogriseolus* subsp. No. 1894	Jurkat T cells Jurkat T-cells, A549, HCT-116, Capan-1	[13] [13,99]
**116**	*S. lusitanus* SCSIO LR32	MDA-MB-435, MDA-MB-231, NCI-H460, HCT-116, HepG2, MCF10A	[30]
**117**	*Streptomyces* sp. M268	HL-60, A549, BEL-7402	[77]
**118**	*Micromonospora* sp.	L1210, MOSE, MOE	[78]
**119**	*Streptomyces* sp. SS13I	PC3, H1975	[79]
**120**	*Streptomyces* sp. HN-A124	A2780	[80]
**121**	*Streptomyces* sp. OC1610.4	MCF-7, MDA-MB-231, BT-474, MDA-MB-231	[81]
**122**	*Streptomyces* sp. XS-16	MDA-MB-231, K562, ASPC-1, H69AR, H69	[83]
**123**–**128**	*Actinomadura* sp. KD439	P388	[84]
**129**	*Streptomyces* sp. SUD119	SK-HEP1	[85]
**130**	*Streptomyces* sp. SUD119	HCT-116, MDA-MB-231, SNU638, A549, SK-HEP1	[85]
**131**, **132**	*Streptomyces* sp. HDN15129	HL-60, K562, SH-SY5Y, BEL-7402, U87, ASPC-1, HCT-116	[86]
**133**	*Streptomyces* sp. CNZ-748	PMP501-1, PMP457-2	[87]
**134**, **135**	*Streptomyces* sp. CNZ-748	PMP501-1, PMP457-2, ABX023-1, C09-1	[87]
**136**, **137**	*Streptomyces* sp. M7_15	SJCRH30	[88]
**138**, **141**	*Amycolatopsis* sp. HCa1	HeLa	[89]
**140**	*Amycolatopsis* sp. HCa1	SPC-A-1, HeLa	[89]
**142**	*Amycolatopsis* sp. HCa1	SGC-7901, HeLa	[89]
**143**	*Streptomyces* sp. Om-4842	P388	[90]
**144**–**149**	*S. griseorubiginosus* No. Q144-2	VCR-resistant P388	[91]
**150**–**154**	*Streptomyces* sp. KY40-1	PC3, H-460	[93]
**155**	*S. polyformus* sp. nov. YIM 33176 Mutant strain of *S. cyanogenus*	37 different human tumor cells LL/2, MCF-7, NCI-H460	[94] [108,109]
**156**, **157**	*Streptomyces* sp. N05WA963	SW620, YES-4, U251SP, K562, MDA-MB-231, T-98	[95]
**158**	*Streptomyces* sp. N05WA963	SW620, YES-4, U251SP, K562, MDA-MB-231, T-98	[95]
**159**	*Streptomyces* sp.Acta 2930	NIH-3T3, HepG2, HT-29	[96]
**160**	*Streptomyces* sp. PU-MM59	PC3, A549	[97]
**161**–**169**	Mutant strain of *S. cyanogenus*	LL/2, MCF-7, NCI-H460	[108,109]
**164**, **167**, **173**–**175**, **177**	*S. cyanogenus* S-136	MCF-7, MDA-MB-231	[114]
**172**	*S. cyanogenus* S-136	MDA-MB-231	[114]
**181**–**184**	*S. cyanogenus* K62	MCF-7, MDA-MB-231	[115]
**185**, **186**	Mutant strain of *S. lividans* TK24	Several cancer cells	[109,116]
**187**	*S. murayamaensis*	K562	[69,117]
**188**–**191**	*S. pacifica* DPJ-0019 (NRRL 50168)	K562, LNCaP, HCT-116, HeLa	[118]
**193**	Unknown actinomycetes	SF-268, MCF-7, HepG2, A549	[119]
**195**	*Amycolatopsis orientalis* subsp. vinearia	Ehrlich ascites carcinoma	[120]
Antibacterial or antifungal activities			
**1**	*Chainia purpurogena*	Gram-positive bacteria except *M. tuberculosis*	[16]
**2**	*Streptomyces* sp. HB202	*B. subtilis* DSM 347, *B. epidermidis* DSM 20660, *D. hominis* DSM 7083, *K. pneumoniae*, *P. aeruginosa* DSM 50071, *S. aureus* ATCC 12600, *S. aureus*, *S. epidermidis* DSM 20044, *S. lentus* DSM 6672	[17]
**3**, **4**	*Streptomyces* sp. QD01-2	*S. aureus*, *B. subtilis*, *Escherichia coli*, *Candida albicans*	[18]
**5**, **6**	*Streptomyces* sp. QD01-2 *S. gilvotanarens* NRRL 11382	*S. aureus*, *B. subtilis*, *Escherichia coli*, *Candida albicans* *S. aureus* ATCC 6538P, *B. subtilis* No. 10707	[18] [31]
**7**–**9**	*S. fradiae* PTZ0025	*S. aureus*	[19]
**10**	*M. rosaria* SCSIO N160 *S. cellulosae* YIM PH20352 *Streptomyces* sp. XZHG99T *Streptomyces* sp. IB201691-2A	*E. coli* ATCC 25922, *S. aureus* ATCC 29213, *B. thuringiensis* SCSIO BT01, *B. subtilis* SCSIO BS01 *P. cucumerina*, *Alternaria panax*, *F. oxysporum*, *F. solani*, *M. smegmatis*, *S. aureus* *S. carnosus* DSMZ 20501, Erwinia persicina DSMZ 19328 *S. carnosus* DSMZ 20501, *M. smegmatis* DSMZ 43286	[20] [59,60] [61] [62]
**11**	*M. rosaria* SCSIO N160	*E. coli* ATCC 25922, *S. aureus* ATCC 29213, *B. thuringiensis* SCSIO BT01, *B. subtilis* SCSIO BS01	[20]
**12**	*M. echinospora* SCSIO 04089	*S. aureus* ATCC 29213, *B. thuringensis* SCSIO BT01, *B. subtilis* 1064, *M. luteus* SCSIO ML01, MRSA shhs-A1	[21]
**15**	*M. echinospora* SCSIO 04089	*M. luteus* SCSIO ML01,	[21]
**17**	*S. pratensis* KCB-132	A variety of bacteria and fungi	[22]
**18**, **19**	*S. pratensis* KCB-132	*B. cereus*, *C. lagenarium*	[22]
**20**	*S. pratensis* KCB-132	*S. aureus* CMCC 26003	[23]
(–)-**21**	*Streptomyces* sp. KCB-132	*B. cereus* CMCC 32210	[24]
**23**	*Streptomyces* sp. KCB-132	*S. aureus*, *Enterococcus faecium*	[25]
**24**	*S. pratensis* KCB-132	*E. faecium*, *S. aureus*, *K. pneumoniae*, *A. baumannii*, *P. aeruginosa*, *E. species*	[26]
**25**–**27**	*S. ardesiacus* 156VN-095	*B. subtilis* KCTC 1021, *M.s luteus* KCTC 1915, *S. aureus* KCTC 1927	[27]
**28**–**30**	*Streptomyces* sp. BCC45596	*M. tuberculosis*, *P. falciparum*	[28]
**31**	*Streptomyces* sp. BCC45596 *S. cellulosae* YIM PH20352	*M. tuberculosis*, *P. falciparum* *A. panax*	[28] [59,60]
**32**, **33**	*Streptomyces* sp. SCSIO 11594	*E. faecalis* ATCC29212	[29]
**34**	*S. lusitanus* SCSIO LR32	*M. luteus*	[30]
**36**–**39**	*Streptomyces nodosus* MH190-16F3	*S. aureus* FDA209P, *S. aureus*, *M. 1ysodeikticus* IFO 3333, *M. luteus* PCI1001, *B. subtilis* PCI 219	[32]
**41**, **42**	*S. antibioticus* Tü 6040	*B. brevis* DSM30	[34,35]
**43**–**46**	*S. violaceolatus*	*S. aureus* FDA 209P, *B. subtilis* ATCC 6633, *B. cereus* IAM 1729, *M. luteus* ATCC 9341	[36]
**46**	*S. capoamus*	*P. chrysogentrrn* ATCC 10002, *T. mentagrophytes*	[37]
**47**	*S. griseoincarnatus*	*S. aureus* FDA 209P, *M. luteus* ATCC 9341, *B. cereus* IAM 1729	[38]
**48**–**56**	*S. venezuelae* ISP5230	*S. aureus* C622 (ATCC25923), *S. aureus* 305, *S. aureus* BeckerCP8 (ATCC49525), *S. aureus* BeckerLyc12CP336 (ATCC55804), *S. epidermidis* C960 (ATCC14990), *S. epidermidis* C621 (clinical isolate), *B. subtilis* C971 (ATCC6633), *S. aureus* C623(MRSA)	[39]
**57**, **58**	*S. venezuelae* ISP5230	MRSA, *S. warneri*, VRE	[43]
**59**	*Streptomyces* sp. AC113 *Streptomyces* sp. CB01913	*P. aeruginosa* CCM 3955, *S. aureus* CCM 3953, *E. coli* CCM 3988, L. monocytogenes NCTC 4886, *B. subtilis* CCM 2216, *B. cereus* *S. aureus* ATCC 25923, *B. subtilis* ATCC 23857, *M. smegmatis* ATCC 607	[44] [63]
**60**–**61**	*Streptomyces* sp. AC113	*P. aeruginosa* CCM 3955, *S. aureus* CCM 3953, *E. coli* CCM 3988, *L. monocytogenes* NCTC 4886, *B. subtilis* CCM 2216, *B. cereus*	[44]
**62**–**64**	*Streptomyces* sp. Acta 3034	*B. subtilis*	[51]
**65**–**67**	*Saccharopolyspora* BCC 21906	*M. tuberculosis*	[52]
**68**	Saccharopolyspora BCC 21906 *Streptomyces* spp. GW19/1251 and GW10/1118	*M. tuberculosis* *B. subtilis*, *S. viridochromogenes* Tü57, *S. aureus*, *E. coli*	[52] [134]
**69**–**71**	*S. salbus*	MRSA, *B. subillis* RM125	[53]
**73**	*S. chattanoogensis* L10 (CGMCC 2644)	*B. subtilis* ATCC 67736	[54]
**75**	*S. blastomycetica* F4-20	*Valsa mali*, *C. orbiculare*, *F. graminearumat*	[56]
**76**	*S. bulliGJA1*, *Gardenia jasminoides*	MRSA	[57]
**78**, **79**	*D. Aptenodytes* NJES-13T	*S. aureus*, *B. subtilis*, *C. albicans*	[58]
**80**	*S. cellulosae* YIM PH20352	*P. cucumerina*, *A. panax*, *F. oxysporum*, *F. solani* with	[59,60]
**81**	*S. cellulosae* YIM PH20352	*P. cucumerina*, *A. panax*	[59,60]
**82**	*S. lusitanus* OUCT16-27	*E. faecium*, *E. faecalis*, *S. aureus*	[126]
**88**, **89**	*Streptomyces* sp. IB201691-2A	*S. carnosus* DSMZ 20501, *E. persicina* DSMZ 19328, *M. smegmatis* DSMZ 43286	[62]
**90**, **92**	*Streptomyces* sp. CB01913	*S. aureus* ATCC 25923, *B. subtilis* ATCC 23857, *M. smegmatis* ATCC 607	[63]
**93**	*S. lividans* TK23 *S. albus* J1074	*S. aureus*, *C. albicans* *B. subtilis* DSM 1092, *M. luteus* DSM 20030	[64] [140]
**94**, **95**	*N. lurida*	Gram-positive bacteria	[65]
**96**	*S. matensis* A-6621	*S. aureus* 209P-JC, Sepidermidis IID 866, *E. faecium* ATCC8043, *B. cereus* S 1101, *B. subtilis* ATCC6633	[67]
**97**–**99**	*Nocardia* sp. IFM 0089	*S. aureus* 209P, *S. aureus* MRSAIFM 62971, *M. smegmatis* ATCC607, *M. luteus* IFM 2066	[68]
**100**–**103**	*S. murayamaensis*	Gram-positive bacteria	[70]
**104**	Unknown actinomycetes	MRSA, VRE	[71]
**106**	*S. aureofaciens* CCM 3239	*B. subtilis*, *S. aureus*	[72]
**116**	*S. lusitanus* OUCT16-27	*E. faecium*, *E. faecalis*, *S. aureus*	[126]
**136**	*S. griseus* NTK 97	*B. subtilis* DSM 10, *S. aureus* DSM 20231	[147]
**140**–**142**	*Nocardia*. sp. M-53	*Bacillus*, *Staphylococcus*, *Micrococcus*, *Cotrnebacterium*, *Mycobacteriu*	[131]
**196**	*Streptomyces* sp. B6219	*S. viridochromogenes* Tü57	[121]
**197**–**199**	*S. espanaensis* AN113	*B. subtilis*, *E. faecium*, *Xanthomonas* sp. pv. Badrii	[122]
**200**, **201**	*S. pratensis* NA-ZhouS1’s	*P. aeruginosa* CMCC (B) 10104, MRSA, *K. pneumonia* CMCC (B) 46117, *E. coli* CMCC (B) 44102, *B. subtilis* CMCC (B) 63501	[123]
**202**–**204**	*Nocardiopsis* sp. HB-J378	MRSA	[124]
**205**	*Nocardiopsis* sp. HB-J378	MRSA, VRE, *B. cereus*	[125]
**206**, **207**	*Nocardiopsis* sp. HB-J378	MRSA	[124]
**208**–**210**	*Saccharothrix* sp. D09	*H. pylori*	[127]
**211**, **212**	*Streptomyces* sp. BHB-032	*S. aureus* CMCC 26003, *Nocardia*, *B. cereus* CMCC 32210, *B. subtilis* CMCC 63501	[128]
**213**–**215**	*M. rosaria* SCSIO N160	*E. coli* ATCC 25922, *S. aureus* ATCC 29213, *B. thuringensis* SCSIOBT01, *B. subtilis* SCSIO BS01, *C. albicans* ATCC 10231.	[129]
**216**	*M. rosaria* SCSIO N160 in a heterologous host *S. coelicolor* YF11	*K. pneumoniae* ATCC 13883, *A. hydrophila* ATCC 7966, *S. aureus* ATCC 29213	[130]
**217**, **218**	*Streptomyces* sp. DSM 4769	*S. aureus* H 503, *S*. *pyogenes*	[132]
**219**, **220**	*Streptomyces* sp. WK-6326	*B. subtillis*, *M. luteus*, *B. subtillis*, *S. aureus*, *M. luteus*, *M. smegmatis*	[133]
**221**	*Streptomyces* spp. GW19/1251 and GW10/1118	*B. subtilis*, *S. viridochromogenes* Tü57, *S. aureus*, *E. coli*, *Chlorella vulgaris*, *C. sorokiniana*	[134]
**222**	*Streptosporangium* sp. Sg3	*M. luteus* ATCC 9314, *B. subtilis* ATCC 6633, *S. aureus* CIP 7625, *L. monocytogenes* CIP 82110, *M. smegmatis* ATCC 607	[135,136]
**223**	*Streptomyces* sp. MK844-mF10	*S. aureus*, *B. subtilis*	[137]
**224**	*Kitasatospora* sp.	*S. aureus* Newman, *P. anomala*, *M. hiemalis*, *E. coli* ToIC	[139]
**225**	*S. albus* J1074	*B. subtilis* DSM 1092, *M. luteus* DSM 20030	[140]
**226**	*S. albus* J1074	*S. aureus* Newman	[140]
**227**, **228**	*Streptomyces* sp. KMC004	*M. luteus*, *E. hirae*, MRSA	[141]
**229**–**232**	*Actinoallomurus* sp. ID145698	*S. aureus* ATCC 6538P, *S. pyogenes* L49, *E. faecalis* L560, *E. faecium* L569	[142]
**233**, **234**	*Actinobacterium* PAL114	*M. flavus* ATCC 9314, *L. monocytogenes* ATCC 13932	[143]
**235**	*S. fimbriatum*	*S. epidermidis* ATCC 12228, MRSA, *S. aureus* ATCC 25923	[144]
**236**	*Actinomycetes* RI104-LiC106	*M. luteus*	[145]
**237**–**240**	*Streptomyces* sp. TK08046	*S. parasitica*	[146]
**241**	*Streptomyces* sp. TK08046	*S. parasitica*, *S. aureus*, *B. subtilis*, *D. pulexwith*	[146]
**242**	*S. tsusimaensis* MI310-38F7	*S. aureus* Smith, *S. aureus* MS9610 (multi-resistant), *M. luteus* PCI 1001, *B. subtilis* NRRLB-558	[148]
**243**	*Actinomadura* sp. RB29	VRE, *M. vaccae*	[149]
**244**, **245**	*S. indonesiensis *DSM41759	MRSA	[150]
Enzyme inhibitory activities			
**1**	*C. purpurogena*	Dopamine S-hydroxylase inhibition	[16]
**42**	*S. coelicolor* YF11 M1152 *S. antibioticus* Tü 6040	DNA gyrase inhibition	[167]
**192**–**194**	Unknown actinomycetes	α-glucosidase inhibition	[119]
**254**	*Streptomyces* sp. KCB15JA014	IDO1 inhibition	[155]
**255**	*Streptomycete* Acta 1362	PTP1B inhibition	[156]
**266**	Unknown actinomycetes	Tyrosine hydroxylase inhibition, dopamine β-hydroxylase inhibition, tryptophan 5-mono-oxygenase inhibition	[163,164,165]
**267**, **268**	Actinomyces MK290-AF1,	FPTase inhibition	[166]
**269**	*S. coelicolor* YF11 M1152 *S. antibioticus* Tü 6040	DNA gyrase inhibition	[167]
**270**, **273**	*Streptomyces* sp. DSM 17045	Antagonize rosiglitazone-induced peroxisome PPAR-γ activation	[168]
**274**–**276**	High-throughput screening of microbial	IDO1 inhibition	[169]
Other activities			
**34**	*Streptomyces* sp. #AM1699	Nitric oxide inhibition	[160]
**74**	Mutant strain of *S. chattanoogensis* L10 (CGMCC 2644)	Antioxidant activity	[55]
**96**	*S. matensis* A-6621	Platelet aggregation inhibition	[170]
**143**	*Streptomyces* sp. P294	Platelet aggregation inhibition	[36]
**208**	Unknown actinomycetes	Nitric oxide inhibition	[128]
**213**	*M. rosaria* SCSIO N160	Antioxidant activity	[129]
**219**, **220**	*Streptomyces* sp. WK-6326	IL-4 inhibition	[133]
**242**	Unknown actinomycetes	*P. burgneri* inhibition	[162]
**246**	*A. heciospongiae* EG49	*T. brucei* brucei inhibition	[151]
**247**, **248**	*A. heciospongiae *EG49	Antioxidant activity	[152]
**249**–**252**	*Actinokineospora* sp. EG49 with *Rhodococcus* sp. UR59	Antimalarial activity	[153]
**253**	*Actinokineospora* sp.	*T. brucei* TC221 inhibition	[154]
**256**	*S. griseus*	Glutaminergic agonist	[157]
**257**	*Streptomyces* sp. KCB15JA151	Cell proliferation inhibition	[158]
**258**, **259**	*Streptomyces* sp. KIB-M10	Human T-cell proliferation inhibition	[159]
**260**, **261**	*Streptomyces* sp. #AM1699	Nitric oxide inhibition	[160]
**262**, **263**	Unknown actinomycetes	*P. falciparum* K1 inhibition	[161]
**264**	*Streptomyces* sp. P294	*P. burgneri* inhibition	[36]
**265**	*Streptomyces* sp. DSM 4769	DNA viruses Herpes simplex I and II	[132]
**277**–**279**	*S. matensis* A-6621	Platelet aggregation inhibition	[170]
**280**, **281**	*Streptomyces* sp. P371	Inhibitory activity against pentagastrin-stimulated acid secretion, protective activity against HCl/ethanol- and indomethacin-induced gastric lesions	[171]
**280**	*Streptomyces* sp. P371	CCK B/gastrin receptor antagonist	[172]
**282**, **283**	*Streptomyces* sp.	SH-SY5Y neuroblastoma cells protection	[173]

## 3. Biosynthesis of Landomycins

Landomycins are one of the best-known subgroups of the angucyclines/angucyclinones, with glycosylation at C8 and hydroxylation at C11 positions [10]. Microbial fermentation, heterologous expression of biosynthetic gene clusters (BGCs), and modification of culture conditions have resulted in the identification of over 30 landomycins. A total of 20 active landomycins (**161**–**167**, **170**–**175**, and **176**–**184**) are involved in this review. Landomycins were first isolated from the fermentation broth of *S. cyanogenus* S136 in 1990 [103], including the hexasaccharidal landomycin A (**164**), the pentasaccharidal landomycin B (**165**), and the disaccharidal landomycin D (**166**). Landomycin A BGC (lan) from *S. cyanogenus* S136 [174], landomycin E BGC (lnd) from *S. globisporus* 1912 [175], and lnd-like BGC [176], identified in the metagenomic DNA, are related to the landomycin biosynthetic pathway. The homologous genes that relate to the biosynthesis of the basic skeleton of the landomycins are almost the same in the three pathways, while there are some differences in details. For example, the putative hexose synthase genes lanZ2, glycosyltransferase lanGT3, and TetR-like repressor gene lanK in the landomycin A BGCs are absent in the landomycin E BGCs, while prx, lndW-W2, and lndY-lndYR, related to the regulation and export of compounds, are missing in the landomycin A BGCs.

**Figure 12:**
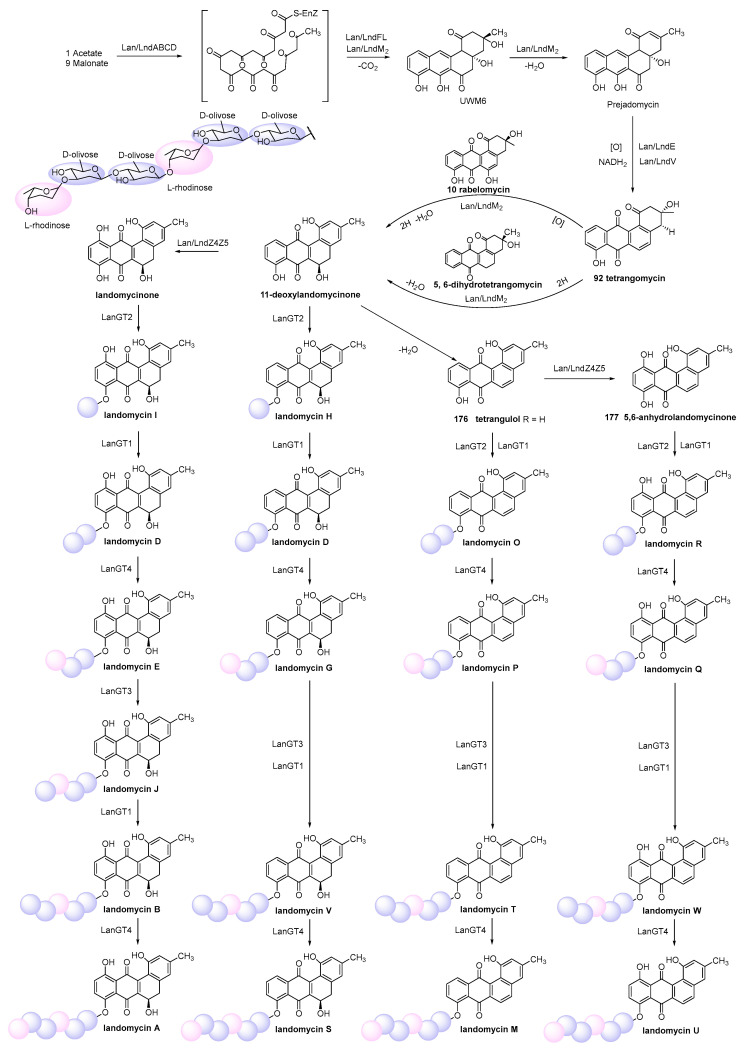
Biosynthesis of landomycins.

Isotope labeling experiments confirmed the origins of the carbon and oxygen atoms in the landomycin angucyclic scaffold [104]**.** The carbons, together with two oxygen atoms at the C-1 and C-8 positions, were proved to originate from acetate, while the oxygen atoms at C-7 and C-12 were found to originate from molecular oxygen. The functions of genes in the BGCs were elucidated by analyzing the products of mutants in which specific genes were inactivated [177]. Products encoded by lanA to lanD are typically found in the biosynthesis of aromatic polyketide, indicating that the genes are used for the synthesis of ketoacyl, the extension of the carbon chain, the transport of acyl groups, as well as the reduction of the ketone group, respectively. The biosynthesis of landomycin involved a decaketide intermediate (Figure 12), which needs to undergo further cyclization, aromatization, oxidation, and reduction to establish the backbone of landomycinone. LanL (homolog of lndL) was suggested to relate to the first cyclization–aromatization during the biosynthesis of landomycins, while lanF (homolog of lndF) was proposed to catalyze the formation of the third and the fourth ring. The products of lnd/lanF and lnd/lanL are also homologous to other cyclases found in type II polyketide gene clusters [177].

Genetic analysis and in vitro assays showed that the tetracyclic framework needs to undergo a cascade of hydrolysis and decarboxylation to form UWM6 after the cyclization. Oxygenases and reductases encoded by lan/lndZ4, Z5, E, M2, and V were proposed for the conversion of UWM6 [178]. Dehydration catalyzed by lan/lndM2 leads to the production of prejadomycin (2,3-dehydroUWM6). C-12 oxygenase encoded by lan/lndE, the first oxygenase in the pathway, could transform prejadomycin to tetrangomycin (**92**) [179]. The 6-keto group was reduced by lan/lndV to form 11-deoxylandomycinone, and lanV also contributed to the aromatization of the landomycin angucyclic scaffold [179]. The inactivation of lndM2 in *S. globisporus* 1912 and the feeding of the intermediate showed that there were at least two paths for tetrangomycin (**92**) to convert to 11-deoxylandomycinone (**181**) [180]. LndM2 catalyzes the hydroxylation at the C-6 position of **92** to yield rabelomycin (**10**), then catalyzes the reduction of the C5–C6 double bond and 2,3-dehydration of **10** to yield **181**. Alternatively, lndM2 reduces the C5–C6 double bond, which converts **92** to 5,6-dihydrotetrangomycin, and then the oxygenation at C-6 and aromatization also generates **181**. LanZ4 and LanZ5 are related to the hydroxylation of the C-11 position. Because of broad substrate specificity, hydroxylation can occur at different stages of glycosylation and independent of the length of the sugar chain [178]. In the presence of multiple substrates, what is the preferred substrate still needs further investigation.

Hexasaccharidal landomycin A is composed of a repetitive sequence of NDP-D-olivose and NDP-L-rhodinose, and four glycosyltransferase genes (lanGT1, lanGT2, lanGT3, and lanGT4) are responsible for six glycosyl transfer steps. The functions were elucidated by expressing individual genes, isolating the products and intermediates, and feeding the intermediates back to the knockout mutant strains [174]. LanGT2 catalyzes the glycosylation step at the C8 position with NDP-D-olivose to produce landomycin H [181]; when flavin reductase LanZ4 and bifunctional oxygenase-dehydratase LanZ5 are functioning, C-11 oxidation can convert landomycin H into landomycin I (**162**). The remarkable feature of LanGT1 and LanGT4 is that they are used twice during the hexasaccharidal landomycin biosynthesis [102]. LanGT1 encodes a D-olivosyltransferase, which is responsible for the attachment of the D-olivose moiety as the second and fifth sugars, converting landomycin I (**162**) to landomycins D (**166**) and B (**165**). LanGT4 is responsible for the attachment of the L-rhodinose moiety (the third and sixth sugars) to produce landomycins E (**161**) and A (**164**). LanGT1 and lanGT4 display a relaxed substrate specificity toward the sugar acceptor substrates. LanGT3 is related to the attachment of the fourth sugar (D-olivose) moiety to yield landomycin J (**163**) [182]. Based on the functions of LanGTs, their unbalancing may lead to the accumulation of specific products. LanGTs can also convert landomycin H directly to landomycins F (**178**), G, V (**174**), and S (**171**) without the oxidation of the C11 position. Dehydration of 11-deoxylandomycinone (**181**) yields tetrangulol (**176**), and oxidation catalyzed by lanZ4/lanZ5 converts tetrangulol (**176**) to 5, 6-anhydrolandomycinone (**177**). Compound **176** could be catalyzed by LanGTs to form landomycins O (**180**), P, M (**179**), and T (**172**), while the glycation products of 5, 6-anhydrolandomycinone were landomycins R (**170**), Q, U (**173**), and W (**175**), respectively (Figure 12).

There are also landomycins [103] wherein the order of L-rhodinose and D-olivose in the sugar chain is different from the landomycins in Figure 12, or the D-olivose is replaced with D-amicetose [115]. Some biosynthetic genes are involved in the export of landomycins. As substances with antibacterial and cytotoxic activity, landomycins are also toxic to their producer. The detoxification mechanism may be related to the effluence of the compounds. LanJ encodes a proton-dependent antiporter protein in *S. cyanogenus* S136. Overexpression of lanJ confers resistance to landomycin A (**164**) and increased accumulation of landomycin with shorter glycoside chains. The TetR-type repressor gene lanK shares the common promoter lanKJp with lanJ and negatively regulates the expression of lanJ [183]. Binding of landomycins to lanK relieves its inhibition and thus triggers the biosynthesis and export of landomycins. lndW has been identified at the end of the landomycin BGCs, and it encodes an ATP-binding subunit of the ABC transporters protein, which is related to the resistance against landomycin [184].

## 4. Discussion

**Figure 13:**
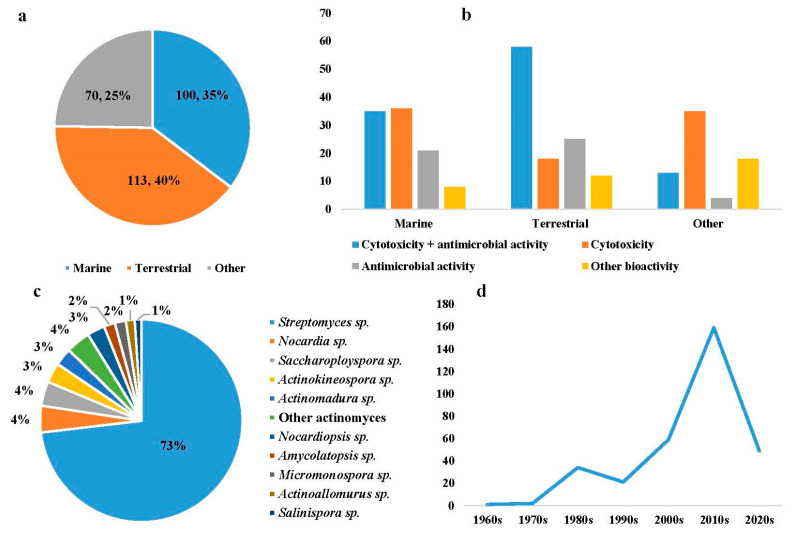
(**a**) Proportion of angucyclines/angucyclinones from different sources. (**b**) Active types of angucyclines/angucyclinones from different sources. (**c**) Producer of bioactive angucyclines/angucyclinones. (**d**) The discovery time of active angucyclines/angucyclinones.

This review covers 283 angucyclines/angucyclinones discovered from 1965 to 2023 with various bioactivities. Marine and terrestrial microorganisms have made nearly equal contributions to the production of these bioactive compounds, affording 100 (35%) and 113 (40%) of them, respectively (Figure 13a). The bioactivities of the compounds are related to their sources. Sixty-five percent of the marine-derived angucyclines/angucyclinones have been reported to show only one type of bioactivity (cell toxicity, antibacterial activity, enzyme activity, anti-inflammation, etc.). Only 35% of the marine-derived compounds show both cytotoxicity and antimicrobial activity, while this figure is 51% for terrestrial-derived angucyclines/angucyclinones (Figure 13b). *Streptomyces* undoubtedly is the most important producer of bioactive angucyclines/angucyclinones (73%), and the gene cluster related to angucyclines/angucyclinones’ biosynthesis in *Streptomyces* has been described in detail in previous reviews [10]. In the future, more angucyclines/angucyclinones might be obtained through gene knockout or the activation of silent genes. The genera *Nocardia* and *Saccharoployspora* follow closely behind, producing 4% of the total included compounds (Figure 13c). Taking 10 years as a statistical unit, the largest number of these molecules was found in the 2010s (Figure 13d), which might relate to the development of separation and structural identification technologies in the 21st century as well as to the increasing number of bioassays. Therefore, it is reasonable to predict that more bioactive novel angucyclines/angucyclinones will be discovered, or new bioactivities might be found for known compounds, in the next ten years.

**Figure 14:**
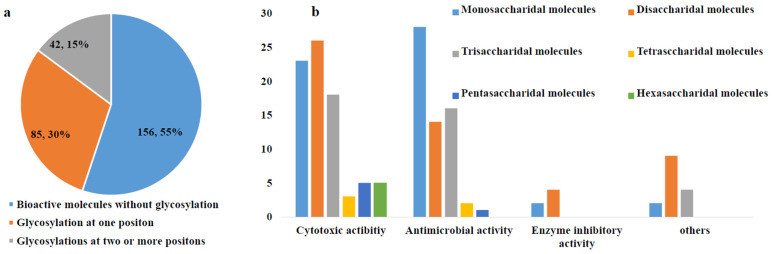
(**a**) Proportion of glycosylated angucyclines/angucyclinones. (**b**) Active types of angucyclines/angucyclinones with different lengths of oligosaccharide chains.

In addition to landomycins, which are characterized by glycosylation at the C8 position, angucyclines/angucyclinones like urdamycins, saquayamycins, and sangkocyclines also can be glycosylated. The number, type, and order of sugars as well as the position of glycoside chains are variable. Among the 283 active angucyclines/angucyclinones collected in this review, 127 compounds contain glycoside chains in their chemical structures, accounting for 45% of the total number. Furthermore, 42 compounds contain 2 glycoside chains, accounting for 15% of the total angucyclines/angucyclinones and 33% of the glycosylated ones (Figure 14a). Disaccharidal members account for the biggest number among all the glycosylated angucyclines/angucyclinones, followed by monosaccharidal and trisaccharidal ones. Disaccharidal angucyclines/angucyclinones tend to display cytotoxic activities, while monosaccharidal ones tend to show antibacterial activities, suggesting that the number of sugars in the glycoside chains may be related to bioactivities (Figure 14b). For example, landomycins with glycosylation at the C8 position display cytotoxicity only. Some researchers believe that most landomycins with bioactivities have long oligosaccharide chains [114,115], while others believe that the cytotoxicity does not increase simultaneously with the elongation of the chain [108,109]. Further research is thus needed to investigate the effects between the oligosaccharide chains and bioactivities of landomycins and the relationship between oligosaccharide chains and glycosylated angucyclines/angucyclinones.

International Whole Genome Sequencing efforts and comparative bioinformatics studies have revealed that the biosynthetic potential of existing microorganisms has not been fully exploited [185,186], and translating genetic blueprints into single compounds remains a significant scientific challenge. Angucyclines/angucyclinones were first discovered in the 1960s and have been a hot topic in the field of drug discovery due to their diverse chemical structures and biological activities. With the rapid development and the application of microbial isolation techniques such as in situ culture [187], analytical techniques like molecular network analysis [188], culturing techniques such as simulated ecological cultivation [149], heterologous gene expression [64], metal stress induction [123], as well as isolation techniques like activity-guided isolation [189] and high-throughput screening [190], the current strategies for discovering microbial natural products have become more specific and efficient compared with traditional methods. This has further enriched the structural diversity of the angucyclines/angucyclinones. Additionally, Global Natural Products Social Molecular Networking (GNPS) has been used widely to analyze the culture extracts of microorganisms and elucidate the important intermediates in the biosynthesis of metabolites [149]. At the same time, the halogenation or substitution with other heteroatoms could modify and enhance the activities of the compounds, and the synthetic derivatization could expand the diversity of molecular structures. All the studies are important to the discovery of more angucyclines/angucyclinones with novel structures and activities.

The biosynthesis toward skeleton and glycosylation of landomycins has been well established, while the functions of genes related to regulation and export of landomycins (such as prx, lndI, lanK, lanJ, lanW, etc.) still need further investigation. At the same time, the differences in biosynthetic pathways between landomycin and other angucyclines/angucyclinones (such as urdamycins, saquayamycins, langkocyclines) are also valuable for further study.

## 5. Conclusions

Endophytic microorganisms, particularly endophytic actinomycetes, have long been regarded as an important source of leading compounds of drugs with broad biological activities [191]. One class of secondary metabolites from microorganisms, angucyclines/angucyclinones, have always been a focus of drug development research due to their unique molecular structures and favorable biological activities. The difference in living environments between marine and terrestrial actinomycetes has led to the isolation of many new microorganisms and the discovery of novel compounds, while research on the isolated microorganisms is still ongoing.

The methods mentioned in this paper for discovering new compounds are also applicable to the discovery of other types of microbial natural products, providing valuable references for finding new natural molecules with diverse structural features. This review summary on the structure and source of active angucyclines/angucyclinones can also provide researchers with new research directions and inspirations for transformation.
